# A feature-based qualitative assessment of smoking cessation mobile applications

**DOI:** 10.1371/journal.pdig.0000658

**Published:** 2024-11-21

**Authors:** Lydia Tesfaye, Michael Wakeman, Gunnar Baskin, Greg Gruse, Tim Gregory, Erin Leahy, Brandon Kendrick, Sherine El-Toukhy

**Affiliations:** 1 Division of Intramural Research, National Institute on Minority Health and Health Disparities, National Institutes of Health, Rockville, Maryland, United States of America; 2 ICF, Reston, Virginia, United States of America; Iran University of Medical Sciences, ISLAMIC REPUBLIC OF IRAN

## Abstract

Understanding users’ acceptance of smoking cessation interventions features is a precursor to mobile cessation apps’ uptake and use. We gauged perceptions of three features of smoking cessation mobile interventions (self-monitoring, tailored feedback and support, educational content) and their design in two smoking cessation apps, Quit Journey and QuitGuide, among young adults with low socioeconomic status (SES) who smoke. A convenience sample of 38 current cigarette smokers 18-29-years-old who wanted to quit and were non-college-educated nor currently enrolled in a four-year college participated in 12 semi-structured virtual focus group discussions on GoTo Meeting. Discussions were audio recorded, transcribed verbatim, and coded using the second Unified Theory of Acceptance and Use of Technology (UTAUT2) constructs (i.e., performance and effort expectancies, hedonic motivation, facilitating conditions, social influence), sentiment (i.e., positive, neutral, negative), and app features following a deductive thematic analysis approach. Participants (52.63% female, 42.10% non-Hispanic White) expressed positive sentiment toward self-monitoring (73.02%), tailored feedback and support (70.53%) and educational content (64.58%). Across both apps, performance expectancy was the dominant theme discussed in relation to feature acceptance (47.43%). Features’ perceived usefulness centered on the reliability of apps in tracking smoking triggers over time, accommodating within- and between-person differences, and availability of on-demand cessation-related information. Skepticism about features’ usefulness included the possibility of unintended consequences of self-monitoring, burden associated with user-input and effectiveness of tailored support given the unpredictable timing of cravings, and repetitiveness of cessation information. All features were perceived as easy to use. Other technology acceptance themes (e.g., social influence) were minimally discussed. Acceptance of features common to smoking cessation mobile applications among low socioeconomic young adult smokers was owed primarily to their perceived usefulness and ease of use. To increase user acceptance, developers should maximize integration within app features and across other apps and mobile devices.

## Author summary

Smoking cessation mobile apps have features to help people stop smoking. The most common features include allowing users to track their smoking behavior and triggers, to receive tailored support, and to access information on how to quit smoking. We asked 38 non-college-educated individuals who smoke about their opinions of these features as presented in two cessation apps, QuitGuide and Quit Journey. The first has been publicly available since 2010, and the second is a new app that our team has developed. People thought the features of self-monitoring, tailored feedback and support, and educational content were useful and easy to use. Specifically, they thought these features made tracking their smoking behavior easy and reliable, accommodated their individual needs, and allowed easy access to quitting information. They had concerns about the burden imposed on users to input information into the app, that the timing of tailored support might not match the times when they need support, and that the quit information was already known. We will use these suggestions to improve Quit Journey. Understanding people’s opinions about features of smoking cessation apps is important because people are inclined to use technologies if they see them as useful and easy to use.

## Introduction

Smoking cessation mobile applications are promising tools for supporting cessation [1]. They are comprised of single or multiple interventional features that are designed to affect behavioral change. These features are considered “active ingredients” or behavioral change techniques (BCTs) that intervene on the mechanisms of action or causal pathways of smoking behavior [2,3]. BCTs are rooted in various theoretical frameworks (e.g., social cognitive theory) [4,5]. The translation of the theoretical underpinnings of BCTs to digital platforms involves a design process, often expert-led, that results in different informational structure and aesthetic display of features across smoking cessation mobile apps. The number, type, and aesthetic quality of features included in smoking cessation apps vary. However, several features are foundational given how commonly used they are. These features include self-monitoring, tailored feedback and support, and educational content [6,7].

Self-monitoring is commonly utilized in digital behavioral health change interventions [8–12]. It is the periodic self-recording or passive capturing of a behavior and its outcomes to enhance self-management [13]. In adaptive health interventions, self-monitoring is aimed at providing continuous feedback on health goals and at triggering support whenever and wherever support is needed [14]. Underlying self-monitoring is a self-regulatory mechanism that directs purposeful behaviors [15]. Self-monitoring is a BCT recommended for smoking cessation interventions to elicit cessation [16]. In smoking cessation apps, self-monitoring is often either self-reported (e.g., recording cigarettes smoked) [17] or system-generated (e.g., calculating money saved by not smoking) [18]. Smoking-related physiological parameters (e.g. exhaled carbon monoxide levels) are also candidates for self-monitoring [10] although biomonitoring remains limited in existing cessation apps as is evident by the volume of calculator and calendar/tracker smoking cessation apps versus those that incorporate physiological monitoring [19].

Tailoring, another staple of health interventions, refers to “any combination of information and behavior change strategies intended to reach one specific person, based on characteristics that are unique to that person, related to the outcome of interest, and derived from an individual assessment”[20]. Tailoring affords mobile interventions the ability to cater feedback and support to each individual and their health needs [21]. Evidence shows that tailored health interventions can be more effective than their generalized, one-size-fits-all counterparts [22–24]. Virtually all theoretical frameworks state that behavioral change is dependent on various factors that vary across individuals (e.g., attitudes, skills) and within individuals (e.g., their readiness to adopt or cease a behavior over time) [25]. Specific to smoking cessation, tailored feedback and support (e.g., provide feedback on behavior) are recommended BCTs that facilitate cessation [16]. Smoking cessation apps can approach tailoring in various ways [26]–from basic (e.g., allowing users to set a quit date and list personal reasons for quitting) [27] to advanced tailoring of treatment timing, type, and dose (e.g., just-in-time adaptive stress management strategies to mitigate stress-induced smoking lapses) [28].

Educational content is the backbone of health interventions regardless of the intervention delivery mode (e.g., print, digital). The provision of information is rooted in theoretical frameworks such as the knowledge-attitude-behavior (KAB) model that underscores the importance of relevant knowledge in shaping attitudes, which in turn affects behaviors [29]. Indeed, the inclusion of detailed information in mobile apps has proven to positively affect health behavior [12]. Consistent with BCTs and the clinical guidelines for treating tobacco use and dependence [16,30], cessation apps often include information on tobacco dependence, consequences, and treatment resources (e.g., quitlines, medication) [6,31].

Self-monitoring, tailored feedback, and educational content are perceived as important features among users of smoking cessation apps to achieve cessation and positively influence use [32,33]. However, use of interventional features and their effects on health outcomes differ across populations. Evidence shows vulnerable (vs. affluent) populations have a lower likelihood of using health apps generally or app features specifically [34–36]. For example, a study found that those with lower incomes (vs. those with more economic resources) were less willing to use self-monitoring in diet and fitness mobile apps [35]. Other studies found that provision of information in health interventions had a particular benefit for people who are socioeconomically disadvantaged, possibly because there are greater knowledge gaps among such groups [37]. Studies show that interventions with fewer interventional features were more effective among populations with lower incomes [37], whereas number of features has been associated with better [38] or no difference [39] in outcomes among general populations. Finally, design concepts of health apps, which relate to multiple elements such content input and delivery, navigation, information architecture, typography, and aesthetics of the app, are a necessary determinant of users’ engagement and their seamless interactions with an app [40,41]. However, studies show that some health apps are not designed to accommodate individuals with certain user profiles (e.g., limited health and digital literacy), which can negatively impact user engagement [42].

Capturing users’ perceptions of smoking cessation apps, their features, and their design concepts can inform our understanding of the differential use of interventional features. This is particularly important among high priority populations for smoking cessation efforts. Lower socioeconomic status (SES) is linked with higher prevalence of cigarette smoking across age, race/ethnicity, and U.S. census region [43] People who smoke and are socioeconomically disadvantaged tend to underutilize traditional smoking treatments for several reasons (e.g., lack of access [44], feelings of guilt and shame that accompany admissions of nicotine addiction [45]) but have positive attitudes toward and show interest in mobile app-based cessation support [46,47]. However, there are few mobile interventions that target individuals with low SES [6]. Technology-based interventions targeting disadvantaged populations are also characterized by having low methodological quality and small effects on cessation [48], suggesting a need to engage this target population when developing interventions. Previous studies have cataloged types of smoking cessation apps and features [7,17,19,31]; their alignment with evidence-based nicotine treatment guidelines [19,49,50]; the associations between features’ presence and user engagement and/or quit success [27,51]; and smokers’ general opinions of feature importance/preference [32,33]. There is limited qualitative work on low SES smokers’ perceptions of cessation apps and their features despite the importance of user acceptance as a determinant of use [52]. This qualitative study aimed to gauge perceptions of self-monitoring, tailored feedback and support, and educational content as presented in two smoking cessation apps, QuitGuide, a benchmark app, and Quit Journey, a newly developed app. The study will inform our understanding of the acceptance of individuals with low SES who smoke of these common interventional features and their design as part of the screening and selection of intervention components that are core to the preparation phase of the multi-phase optimization strategy (MOST) for the development and optimization of Quit Journey, a smoking cessation intervention targeting individuals of low SES who smoke [53,54].

## Methods

### Intervention description

Quit Journey is a newly developed mobile app that includes features common to health apps in general and smoking cessation apps in particular (e.g., self-monitoring). Additionally, the app includes new components (e.g., carbon monoxide monitoring). A complete description of Quit Journey appears elsewhere. We used QuitGuide as a template for the three features examined in this study that were included in Quit Journey. QuitGuide is operated and maintained by the National Cancer Institute’s Smokefree.gov Initiative (SFGI) [55]. We selected QuitGuide as a template because it has been available to the U.S. public since 2010 with 195,476 unique downloads since its release and with observed quit rates for those using the app ranging between 3% and 15% [56]. QuitGuide is also often referenced in authoritative smoking cessation resources and used in research on smoking cessation apps (usually as a control or baseline app) [57].

Generally, the three features examined here allow users to self-monitor mood, cravings, and slips; to track the number of smoke-free days, number of cigarettes not smoked, and money and time saved by being smoke free; to receive tailored feedback and support based on slip entries, mood and craving, and user-determined times and locations when/where users anticipate needing support; and to access on-demand educational content that includes guidance on how to quit, benefits of quitting, strategies to handle cravings and withdrawal symptoms, and connections to additional online cessation support resources (i.e., Smokefree.gov) including information about how to access smoking cessation counseling.

### Ethics approval and consent to participate

The study was deemed exempt by the National Institutes of Health Institutional Review Board on 10/11/2019 under Category 2: Research that only includes interactions involving educational tests, survey procedures, interview procedures, or observation of public behavior (§45 CFR 46.10(d)(2)); and Category 3: Research involving benign behavioral interventions (§45 CFR 46.10(d)(3)). The study was deemed exempt by ICF’s Institutional Review Board on 11/19/2019 under Category 2. An amendment was approved by ICF’s Institutional Review Boad on 2/26/2020.

### Participants and Recruitment

We recruited a convenience sample of 38 young adult current smokers, ages 18 to 29 years old. Studies show that individuals who quit smoking at a young age have reduced mortality risks [58–60]. UserWorks, Inc. (Silver Spring, MD) sent candidate participants an email invite and screened those interested in the study for enrollment eligibility over the phone. Participants were not college-educated and not currently enrolled in a 4-year college as an indicator of socioeconomic status [61–63]. Furthermore, participants were willing to quit within six months, did not use non-cigarette combustible tobacco products nor smoking cessation aids, owned a smartphone, and spoke English. Participants received a $150 gift card incentive. Participant recruitment and focus group discussions occurred from 01/2020 to 04/2020. Data saturation did not influence recruitment, and no participants withdrew from the study. Study procedures and recruitment methods have been published elsewhere [64].

### Procedures

We conducted 12 semi-structured, virtual focus group discussions on GoTo Meeting, an online videoconferencing platform. Focus groups were ~1.5 hours long and we conducted dry runs prior to the final 12 focus groups. TG (male), a user experience strategist with training experience in focus groups, moderated the discussions while EL (female), a communications and marketing project director, took notes and acted as a backup moderator. Only the participants and TG, EL, and SEL, a senior investigator on the project (female) who listened in on the discussions, were present during the focus group sessions. Each participant was assigned a unique ID that was used during the discussions and in data analysis.

TG reviewed the consent form and participants verbally consented to participate in the study before the discussions and audio recording began. TG emphasized having no affiliation with the sponsors of either mobile app to reduce social desirability bias [65]. There were no prior relationships between the participants and moderators. Discussions were audio recorded and auto transcribed via GoTo Meeting’s built-in auto transcription feature. Transcripts were not returned to participants for feedback. The 32-item consolidated criteria for reporting qualitative research (COREQ) checklist appears in S1 Table [66].

### Moderation Guide

The guide started with questions about the apps’ names and landing pages, followed by the three interventional features examined here as presented in both apps (S1 Note). Questions gauged smokers’ acceptance of each feature, informed by the second Unified Theory of Acceptance and Use of Technology (UTAUT2) [52,67], which outlines factors associated with user acceptance of technology. Questions were identical across all 12 focus groups except for app screenshots shown to participants, with those in focus groups 1 to 8 seeing screenshots from QuitGuide and those in groups 9 to 12 seeing screenshots from Quit Journey. Twelve participants participated in one session for QuitGuide and one for Quit Journey. Noteworthy, the moderation guide gauged participants’ perceptions of other interventional features of smoking cessation mobile applications that are reported elsewhere.

### Analysis

Following a deductive thematic analysis approach [68], we identified initial codes in an exploratory phase of the analysis. Then, we identified salient themes using a codebook that included (a) technology acceptance themes based on UTAUT2 (i.e., performance expectancy, effort expectancy, hedonic motivation, facilitating conditions, social influence) [52,67], (b) sentiment (i.e., positive, neutral, negative) (c) interventional feature (i.e., self-monitoring, tailored feedback and support, educational content), (d) design concept, and (e) suggestions. We also identified relevant themes regarding each app overall, app name, and app landing page and were open to emerging themes (e.g., intent/willingness to use a feature).

LT and MW independently coded the transcripts using ATLAS.ti (version 8, ATLAS.ti Scientific Software Development GmbH). Following a multi-coding approach, themes within a semantic domain were mutually exclusive but themes from other semantic domains could be applied to the same quote. Inter-coder agreement was calculated via ATLAS.ti. The Krippendorf c-alpha binary value was 0.937, indicating strong agreement amongst coders when differentiating relevant from irrelevant content [69]. The Krippendorf cu-alpha values, which indicate the reliability of each semantic domain [69], appear in Table 1 alongside themes and their definitions. Discrepancies in coding were resolved through discussions amongst LT, MW, and SEL.

**Table 1:** Semantic domains, theme definitions, and inter-coder agreement [64].

Semantic domains and theme definitions	Krippendorf’s cu-Alpha
**Technology acceptance**	0.71
Performance expectancy refers to perceived usefulness or helpfulness of smoking cessation mobile applications and their features in achieving desired health goals and behaviors.	
Effort expectancy refers to perceived ease of use or effortfulness with which one can navigate mobile applications and their features and seamlessly integrate them in one’s life.	
Facilitating conditions are factors that can aid or impede the uptake or use of mobile applications or their features. These include individual-related (e.g., skills, predispositions, prior experiences) and technical-related (e.g., infrastructure) factors.	
Hedonic motivation refers to perceived fun, pleasure, or enjoyment (or lack thereof) associated with the use of mobile applications and their features.	
Social influence refers to perceived importance of significant others’ recommendations and approval of using mobile applications and their features.	
**Sentiment**	0.58
Positive sentiment capture statements or remarks that indicate a sense of approval, praise, or certainty about any aspect of smoking cessation mobile applications and their features such as their worthiness, utility and impact, time and effort investment, and compatibility with one’s life.	
Neutral sentiment capture statements or remarks that (a) are neither positive or negative in tone, (b) contain an equal number of positive and negative remarks, or (c) are conditional (i.e., positive in nature but dependent on the presence or absence of another factor).	
Negative sentiment capture statements or remarks that indicate a sense of disapproval, criticism, or skepticism about any aspect of smoking cessation mobile applications and their features such as their worthiness, utility and impact, time and effort investment, and compatibility with one’s life.	
**Interventional features**	0.88
Self-monitoring: The ability to monitor and record smoking behavior, its antecedents, and consequences when attempting to quit smoking.	
Tailored feedback and support: The delivery of support that is unique to an individual based on user-input regarding smoking behavior, antecedents, progress toward goals, or pre-specified times and locations.	
Educational content: Information related to smoking and smoking cessation, including steps for quitting, quitting strategies, and benefits, among others.	
**Design concepts**: Statements related to user interface, content input and delivery, information structure, navigation, typography, and aesthetics of smoking cessation applications or their features.	-
**Suggestions**: Statements concerned with improvements, modifications, or additions to smoking cessation applications or their features aimed to improve their functionality and/or design.	-
**Emerging themes** ^*^	-
Intent or willingness to use: Statements reflecting intentions or willingness to use smoking cessation applications or their features in the future.	

Multi-value coding of quotes was applied across semantic domain, but themes within a semantic domain were mutually exclusive.

*Themes not defined a priori and thus no inter-coder agreement was calculated for this exploratory part of the analysis.

## Results

Participants were 52.63% female, 42.10% non-Hispanic White, 28.94% non-Hispanic Black, and 15.78% Hispanic adults. Most participants had some college education (47.36%), whereas 26.31% were high school graduates and 10.52% had an associate degree. Sample characteristics appear in Table 2 and detailed participant information in S2 Table. Select illustrative quotes appear below with participant ID attribution and the referenced app (QG for QuitGuide, QJ for Quit Journey).

**Table 2:** Participant characteristics (*N* = 38) [64].

Characteristic	*n* (%)
Sex	
Female	20 (52.63)
Male	18 (47.36)
Race and ethnicity	
NH American Indian or Alaska Native	1 (2.63)
NH Asian, Native Hawaiian or Pacific Islander	3 (7.89)
NH Black or African American	11 (28.94)
Hispanic or Latino	6 (15.78)
NH White	16 (42.10)
Mixed	1 (2.63)
Highest level of education	
Less than high school	3 (7.89)
High school graduate	10 (26.31)
High school equivalent	3 (7.89)
Some college, no degree	18 (47.36)
Two-year associate degree	4 (10.52)
Smoking Frequency	
Every day	30 (78.94)
Some days	8 (21.05)
Quit timeframe	
7 days	11 (28.94)
30 days	22 (57.89)
6 months	5 (13.15)
Smartphone operating system	
Android	21 (55.26)
iOS	17 (44.73)

NH = Non-Hispanic.

### Perceptions of smoking cessation apps interventional features

Across both apps, 312 quotes were feature-related, of which roughly half focused on self-monitoring (*n* = 152, 48.71%), followed by tailored feedback and support (*n* = 112, 35.89%), and educational content (*n* = 48, 15.38%). Discussions focused primarily on feature performance expectancy that ranged from 39.13% to 70.00% of the extracted quotes for each feature. The remaining technology acceptance themes were discussed to a lesser extent: effort expectancy (16.98%), hedonic motivation (16.34%), and facilitating conditions (1.60%). Quotes that captured participants’ sentiment but did not reflect a technology acceptance theme were coded as “not applicable” and constituted 17.62% of quotes. Distribution of quotes by technology acceptance and sentiment appears in Table 3 for QuitGuide and in Table 4 for Quit Journey.

**Table 3:** Distribution of number of quotes by technology acceptance and sentiment toward three interventional features in QuitGuide smoking cessation mobile app.

Themes	Self-monitoring				Tailored feedback and support				Educational content			
	Negative *n (%*)	Neutral *n (%*)	Positive *n (%*)	Total *n* (%)	Negative *n (%*)	Neutral *n (%*)	Positive *n (%*)	Total *n* (%)	Negative *n (%*)	Neutral *n (%*)	Positive *n (%*)	Total *n* (%)
Performance Expectancy	4 (33.33)	3 (15.78)	44 (58.66)	51 (48.11)	3 (42.85)	2 (13.33)	24 (57.14)	29 (45.31)	0 (0.00)	0 (0.00)	7 (77.77)	7 (70.00)
Effort Expectancy	0 (0.00)	6 (31.57)	8 (10.66)	14 (13.20)	1 (14.28)	2 (13.33)	4 (9.52)	7 (10.93)	0 (0.00)	0 (0.00)	2 (22.22)	2 (20.00)
Facilitating Conditions	0 (0.00)	3 (15.78)	1 (1.33)	4 (3.77)	1 (14.28)	0 (0.00)	0 (0.00)	1 (1.56)	0 (0.00)	0 (0.00)	0 (0.00)	0 (0.00)
Hedonic Motivation	5 (41.66)	4 (21.05)	7 (9.33)	16 (15.09)	2 (28.57)	10 (66.66)	1 (2.38)	13 (20.31)	1 (100.00)	0 (0.00)	0 (0.00)	1 (10.00)
Social influence	0 (0.00)	0 (0.00)	0 (0.00)	0 (0.00)	0 (0.00)	0 (0.00)	0 (0.00)	0 (0.00)	0 (0.00)	0 (0.00)	0 (0.00)	0 (0.00)
Not applicable	3 (25.00)	3 (15.78)	15 (20.00)	21 (19.81)	0 (0.00)	1 (6.66)	13 (30.95)	14 (21.87)	0 (0.00)	0 (0.00)	0 (0.00)	0 (0.00)
Total	12 (11.32)	19 (17.92)	75 (70.75)	106 (100)	7 (10.93)	15 (23.43)	42 (65.62)	64 (100)	1 (10.00)	0 (0.00)	9 (90.00)	10 (100)

Column totals add to 100% within each theme, whereas overall row totals add to 100% across a semantic domain.

**Table 4:** Distribution of number of quotes by technology acceptance and sentiment toward three interventional features in Quit Journey smoking cessation mobile app.

Themes	Self-monitoring				Tailored feedback and support				Educational content			
	Negative *n (%*)	Neutral *n (%*)	Positive *n (%*)	Total *n* (%)	Negative *n (%*)	Neutral *n (%*)	Positive *n (%*)	Total *n* (%)	Negative *n (%*)	Neutral *n (%*)	Positive *n %*)	Total *n* (%)
Performance Expectancy	0 (0.00)	0 (0.00)	18 (50.00)	18 (39.13)	2 (50.00)	2 (25.00)	22 (61.11)	26 (54.16)	4 (40.00)	3 (50.00)	10 (45.45)	17 (44.73)
Effort Expectancy	1 (25.00)	1 (16.66)	13 (36.11)	15 (32.60)	1 (25.00)	0 (0.00)	6 (16.66)	7 (14.48)	0 (0.00)	0 (0.00)	8 (36.36)	8 (21.05)
Facilitating Conditions	0 (0.00)	0 (0.00)	0 (0.00)	0 (0.00)	0 (0.00)	0 (0.00)	0 (0.00)	0 (0.00)	0 (0.00)	0 (0.00)	0 (0.00)	0 (0.00)
Hedonic Motivation	0 (0.00)	4 (66.66)	0 (0.00)	4 (8.69)	1 (25.00)	6 (75.00)	1 (2.77)	8 (16.66)	6 (60.00)	2 (33.33)	1 (4.54)	9 (23.68)
Social influence	0 (0.00)	0 (0.00)	0 (0.00)	0 (0.00)	0 (0.00)	0 (0.00)	0 (0.00)	0 (0.00)	0 (0.00)	0 (0.00)	0 (0.00)	0 (0.00)
Not applicable	3 (75.00)	1 (16.66)	5 (13.88)	9 (19.56)	0 (0.00)	0 (0.00)	7 (19.44)	7 (14.58)	0 (0.00)	1 (16.66)	3 (13.63)	4 (10.52)
Total	4 (8.69)	6 (13.04)	36 (78.26)	46 (100)	4 (8.33)	8 (16.66)	36 (75.00)	48 (100)	10 (26.31)	6 (15.78)	22 (57.89)	38 (100)

Column totals add to 100% within each theme, whereas overall row totals add to 100% across a semantic domain.

*Self-monitoring*. Participants had an overall positive sentiment regarding the usefulness of self-monitoring features in helping them quit smoking (QuitGuide: 86.27%, Quit Journey: 100%) such as monitoring aspects of smoking behavior (e.g., slips) and its antecedents (e.g., mood). (S3 Table).

*I really like the mood [tracking] because it’s like every day my mood is up and down, and some days I want a cigarette more than others. So I think this … mood scale is really nice and then you can also see how … you felt that day and … see what you can change as far as your mood and you know to help you not want a cigarette. (P25, QJ)*

The usefulness of self-monitoring features was owed to the reliability of mobile applications, which allow users to identify behavioral patterns over time to stay motivated and accountable.

*I think it would be pretty good … because … keeping track of … what went wrong and … why you went back to smoking or … What is making you smoke is … hard to remember … so an app like this would help a lot. Just keeping track of everything. (P13, QG)*

However, participants remarked that self-monitoring could induce unintended negative reactions when users are unable to maintain positive behavioral patterns over time or do not receive appropriate support when reporting lapses.

*I feel like if … I say [that] I was not smoking for a week and I’d been pressing the … smoke free [button] all week and then I had to press I slipped and it was like … “you’re starting at zero again” and there was no words of encouragement, I might just go “I’m leaving this app, it made me feel bad.” (P22, QG)*

*Tailored feedback and support*. Participants expressed an overall positive sentiment toward usefulness of tailored features of smoking cessation apps generally (QuitGuide: 82.75%, Quit Journey: 84.61%) and toward pre-specified time- and location-based tailored support specifically (S4 Table).

*I think [personalization would be helpful] cause no two people are the same. So, what works for one person, somebody else might need a little adjustment to it for it to work for them or be able to be a better use for them. (P04, QJ)*

*I like the time of day [notifications] the most*. *The location [notifications are] nice*, *and I like that … I like the time [notifications] because certain times make me want to kind of smoke … so [it] would be nice to be able to set that up and have reminders not to*, *or things that I could do during those times*. *(P13*, *QG)*

Skepticism about the usefulness of tailored feedback and support focused on the need for active input from users, which could be “too much.” Additionally, participants questioned the usefulness of notifications given the addictive nature of smoking and the unpredictable timing of cravings.

*… I smoke because it’s like once [a] craving hits, it hits … and then I just pull out a cigarette … If my phone vibrates and reminds me to not smoke [a] cigarette, it may or may not help me put that …cigarette down, because I already have that cigarette in my hand and I’m already addicted to it. (P09, QG)*

*I think the notifications [aren’t] that accurate*, *because you can always have cravings*, *different places*, *different times*. *So*, *it’s not really a specific time*, *you have a craving or a specific place*, *like you can see someone smoking or watch TV and you … have a craving*. *So*, *the notifications … doesn’t really do much*. *(P25*, *QJ)*

*Educational content*. Participants thought the inclusion of cessation information in both apps was useful with 100.00% of QuitGuide quotes and 58.82% of Quit Journey quotes being positive in sentiment (S5 Table). This sentiment was owed primarily to availability of this content on demand.

*I think it’s useful because the app is primarily just for smoking. Whereas if you go like on Google or something you can search [for information] and they give you all these other resources … This is like your own personal journey to … to quit smoking. So, I think it will be helpful. (P16, QJ)*

Negative sentiment about the cessation information’s usefulness centered on the substance of the information presented, which was described as elementary and repetitive particularly for established smokers.

*I honestly think this [cessation information] would be good for … a beginner person that doesn’t really know all these things, because then they can learn. But most of this … is stuff I’ve already read and seen millions of times … The idea of going back to this one section, every time you need help, is kinda slim because … all you’re doing is reading the same thing over and over. (P28, QJ)*

*Other technology acceptance themes*. Beyond feature performance expectancies, participants generally perceived all features to be easy to use.

*I feel like [tracking] could become … part of a daily routine or something like you wake up in the morning, use it once, go again in the evening, and use it again. Like, it doesn’t look too hard to [use] … you don’t really have to … go out of your way to record. So, you can pretty much do it anytime. It’s something … that you can use daily. (P30, QJ)*

*[The cessation information] seems pretty straightforward and easy to comprehend*, *find and use*. *(P10*, *QJ)*

Although most participants agreed that use of features examined here was not engaging nor fun, some stated that enjoyment in using these features was contingent on certain circumstances (e.g., successful quitting) or user predispositions (e.g., having a competitive or analytical nature).

*I think [personalization] might be fun if … you’re a couple of days into quitting smoking and feeling successful. But I think in the times you weren’t feeling successful, it wouldn’t be fun. (P22, QG)*

*When you actually are [seeing] the progress and you’re doing the steps or you can see kind of physically in front of you … how much you’ve cut down*, *I think then it kind of becomes fun … especially if you’re a competitive person [and] you like to see those results in front of you*.. *It kind of gives you confidence … So … I think it would be hard at first*, *because it’s just something you have to get used to*. *(P07*, *QG)*

Participants identified some facilitating conditions to use the features examined here such as personal commitment to quitting smoking.

*But I do think [tracking] would be pretty helpful as far as … it kind of depends on how committed you are to smoking. (P08, QG)*

### Perceptions of design concepts of smoking cessation apps’ features

Participants pointed out several design elements spanning feature pages (*n* = 67), apps’ landing pages (*n* = 44), and overall look and feel (*n* = 20) that could impact their use of a feature (S3–S6 Tables).

Certain design elements elicited negative reactions across both apps. For example, participants indicated use of certain colors (e.g., red) and imagery (e.g., alarm clock icon) carried negative connotations or were symbolically confusing and should be changed.

*I don’t really like the [red] color [of the tracking slips background] either, it [feels] … like you’ve done something wrong like bad or something. And I get that’s what you did, you’ve like messed up but it just seems like it would probably make you feel way worse and not want to get back on track in my opinion. (P13, QG)*

A few participants noted that the educational content section was text heavy and needed visuals (e.g., pictures, videos) to engage users.

*What I dislike is that [the cessation information] might be a lot to read for some people. (P28, QJ)*

### Suggestions to improve smoking cessation apps and their features

Participants’ suggestions were concerned with fully utilizing mobile technologies to improve smoking cessation apps generally (*n* = 78) and pertaining to the three features examined here specifically (*n* = 66) (S7 Table). For example, participants suggested that smoking cessations apps should allow users to self-monitor physiological data.

*I think you guys should add a health section where you can like track your breathing as it gets better, [like] your blood pressure, just health-wise so you can know how much you’re improving by the day. (P25, QJ)*

Other participants suggested integration with other smartphone apps (e.g., Google or Apple Maps, iCalendar) and wearable devices that would allow for passive gathering of data and subsequently the provision of just-in-time support with minimal user input.

*I think it would also be cool if [the app could] connect to something like a Fitbit or whatever. If …you log lapses at … certain locations … it can track your location or time. Maybe … it can have a little area where it’s like “do you need suggestions?” It’s gonna be like … “we’ve noticed that … you tend to … lapse at this time or … this location … [so] … do you need extra help then?”(P11, QJ)*

Participants suggested adding features to smoking cessation apps to potentially increase user acceptance. For example, many participants suggested including a social forum where users can interact with others to form a sense of community.

*I think that’s … the missing ingredient, like that social aspect where you’re connecting with others, that [would] enhance it even more and make it even better. (P27, QJ)*

Design-related suggestions focused primarily on the color palette and the addition of more engaging app elements (e.g. videos, pictures).

*So maybe [adding] little videos … little clips, instead of so many words [for the educational content]. (P34, QJ)*

Results representing participants’ perceptions of Quit Journey and QuitGuide (i.e., app name, app landing page, and app in general) appear in S2 Note and S7 Table.

## Discussion

This study established positive perceptions of three interventional features of mobile smoking cessation interventions as presented in two apps, Quit Journey and QuitGuide, among individuals with low SES who smoke. They perceived self-monitoring, tailored feedback and support, and educational content as both useful and easy to use features, two technology acceptance themes that have been documented to facilitate technology use [70]. Less emphasis was placed on other technology acceptance themes (e.g., social influence) except for individual predispositions that could facilitate or impede the use of app features [52,67]. Features were generally not perceived as fun or engaging, which could potentially be a barrier to sustained use [52,67]. While Quit Journey generally fared better than QuitGuide in its design concepts (S2 Note), participants made suggestions applicable to both apps such as increasing their interactivity, customization capabilities, and integration with other apps and devices. These recommendations align with users’ expectations of mobile health apps, which are important determinants when aiming to increase app use [32,64,71]. Indeed, health apps that fail to meet users’ expectations are perceived as less useful and possibly deleted shortly after they are downloaded [72]. Given the paucity of studies on users’ acceptance of the “active ingredients” of behavioral interventions, especially among vulnerable populations that smoke, this study fills an important gap in smoking cessation mobile intervention research. Alongside establishing the acceptability of Quit Journey’s components and informing its design concepts, implementing users’ recommendations could potentially improve user engagement, and consequently uptake and sustained use, of smoking cessation apps generally.

As part of MOST preparation phase, this study shows that acceptance of common interventional features of smoking cessation apps by individuals with low SES who smoke is promising [53,54]. They tend to underutilize traditional smoking treatments [44,45] but favor technology-based cessation support, including mobile apps [46,47]. Thus, mobile apps could provide an alternative cessation approach for this vulnerable population. However, there are limited feature-level analyses that have explored the linkage between the presence of certain features and health intervention effectiveness [73]. With evidence being mixed on specific features and number of features that improve health intervention outcomes [38,39,74], further research is needed to understand the effect of individual features on outcomes, especially among vulnerable populations [37]. Furthermore, the link between acceptance of interventional features and uptake of health apps in real-life warrants further investigation. For example, while there is limited data on vulnerable populations’ uptake of smoking cessation apps, they are less likely to use health apps in general and certain interventional features (e.g., self-monitoring) in particular compared to their more affluent counterparts [34–36]. This could potentially be attributed to individual-level factors (e.g., limited digital literacy) [75] or app-specific factors with health apps being too basic [50]. In planned pilot and optimization studies, we will assess the efficacy of Quit Journey and its components on cessation outcomes [53].

Participants’ suggestions centered on making the app features smarter such as by enabling tailored real-time support with minimal user input. Offering smoking cessation support for populations with low SES in real time is a promising tactic [24], will likely satisfy users’ high expectations for smartphone apps [64], and aligns with shifts toward just-in-time adaptive interventions that deliver personalized treatment whenever and wherever it is needed [76]. Personalized support with passive sensing and minimal user input could entice smokers to utilize cessation apps because repeated active tracking of data can be seen as tedious or boring [77]. More importantly, user-initiated support and recording of smoking episodes is often low [78]. A recent meta-regression study found that personalized interventions that relied on system-captured data were more effective than those that relied on user-reported data [79]. Furthermore, suggestions related to design concepts of both apps align with design principles for digital platforms [41]. For example, participants recommended that the educational content be presented in a less text-heavy format with the inclusion of appropriate imagery, videos, and icons. This corresponds with prior work finding that users prefer multiple (vs. single) modes of communicating information [32,40]. Preference for use of coherent color schemes and bright colors was also emphasized in our study (S2 Note), which further aligns with literature on what design components are important to app users [40] and accentuates the importance of both the health content and how it is presented. Collectively, these results emphasize that developers of smoking cessation apps (and health apps generally) must fully capitalize on the advancements in mobile technologies and include their target populations in the app design process using human-centered design methods to maximize engagement and sustained use [80].

The pervasiveness of mobile technologies and health apps contextualizes our results. As U.S. smartphone ownership rates are reaching saturation across multiple sociodemographic populations (including populations with low SES) [81], mobile apps are increasing in both availability and popularity with users. As of 2024 there are roughly five million apps available for download across leading app stores (e.g., Google Play, Apple App Store, Amazon Appstore) [82]. Furthermore, a national 2018 survey found that 49.24% of Americans had apps related to health and wellness downloaded on their smart device [83]. This explains the predominance of perceived usefulness of smoking cessation apps features as the primary technology acceptance theme discussed, where participants intuitively underscored the mechanisms by which each feature could serve as a tool to help them alter their smoking behavior. Additionally, consistent with recent evidence [84], none of the barriers commonly outlined in the literature to negatively affect use of mobile technologies among populations with low SES (e.g., privacy concerns, cost, low digital/health literacy) [75] emerged in our results. Conversely, per users’ suggestions, it was evident that participants had unmet expectations regarding how smoking cessation apps should look and perform. Specifically, most participants either explicitly said the features presented were not fun or stated that enjoyment was contingent on certain predispositions (e.g., being competitive). While these high expectations could be attributed to our participants being young adults, it is important that health apps employ different engagement strategies (e.g., gamification [85], reciprocity [86]) especially when the use of smoking cessation apps may be viewed as a reminder of one’s “addiction”. Prior work has shown that users often abandon health apps after minimal usage for reasons including lack of fun or interest [36,87]. Further research is needed to identify and examine the effects of engagement strategies, especially non-financial ones, on sustained use of smoking cessation apps among disadvantaged groups.

This study had several strengths and limitations. The study gauged perceptions of young adults with low SES who smoke on three interventional features as presented in two smoking cessation apps. Our qualitative approach to elicit feature-based perceptions yielded a wealth of information on features common to smoking cessation apps either as standalone interventions or as part of multi-component ones. These results are highly informative, whereby through a human-centered design approach, we ensured the involvement of our target audience in intervention development and evaluation processes of our new smoking cessation app, Quit Journey [80]. As a high priority population for smoking cessation efforts, the educational attainment requirement for participants recruited for this study was a strength. However, we relied solely on educational attainment as an indicator of SES to the exclusion of other indicators such as income and employment [61–63]. Furthermore, our participants were young adults who are often digitally literate [88] and who wanted to quit, thus were not representative of all people who smoke such as older individuals. All focus groups were conducted virtually, which occasionally introduced technical difficulties and could have dampened their perceptions of enjoyment associated with app use based on viewing screenshots of the apps’ pages. Because of scheduling issues, we had more focus groups for QuitGuide than Quit Journey (i.e., 8 vs. 4). The clearcut boundaries between features as presented in the moderation guide were arbitrary. As implemented in both smoking cessation apps examined here, features often overlap. For example, when a user self-records their mood, they receive a tailored support message depending on their mood. The moderation guide relied on general terms (e.g., tracking, personalization, cessation information) when prompting discussions about self-monitoring, tailored support and feedback, and educational content as they are more accessible to non-researchers. We applied the “suggestions” theme to users’ recommendations even if the suggestion was already implemented in the app. However, users focused on big-picture recommendations to improve smoking cessation apps rather than on recommendations to improve specific features. Finally, the intercoder agreement for sentiment was low. However, all inconsistencies were resolved through discussions.

## Conclusions

Self-monitoring, tailored feedback and support, and educational content were perceived to be useful and easy to use features of smoking cessation interventions. Results inform the development of Quit Journey, a multi-component smoking cessation mobile application targeting individuals with low SES smokers who have higher-than-average smoking rates. To increase smoking cessation app acceptance, health professionals should aim to fully capitalize on the capabilities of mobile technologies and employ design elements that can fully meet users’ expectations.

## Supporting information

S1 Note Moderation guide. (DOCX)

S2 Note Perceptions of apps’ names and landing pages. (DOCX)

S1 Table Consolidated criteria for reporting qualitative research (COREQ). (DOCX)

S2 Table Detailed participant characteristics. (DOCX)

S3 Table Themes and illustrative quotes of individuals who smoke on the self-monitoring feature in QuitGuide and Quit Journey. (DOCX)

S4 Table Themes and illustrative quotes of individuals who smoke on the tailored feedback and support feature in QuitGuide and Quit Journey. (DOCX)

S5 Table Themes and illustrative quotes of individuals who smoke on the educational content feature in QuitGuide and Quit Journey. (DOCX)

S6 Table Themes and illustrative quotes of overall perceptions of QuitGuide and Quit Journey, names, and landing pages. (DOCX)

S7 Table Illustrative quotes of suggestions to improve QuitGuide and Quit Journey. (DOCX)

## References

[1] US Preventive Services Task Force. Interventions for tobacco smoking cessation in adults, including pregnant persons: US preventive services task force recommendation statement. JAMA 2021;325(3):265–279. doi: 10.1001/jama.2020.25019 33464343

[2] Michie S, Richardson M, Johnston M, Abraham C, Francis J, Hardeman W, et al. The behavior change technique taxonomy (v1) of 93 hierarchically clustered techniques: building an international consensus for the reporting of behavior change interventions. Ann Behav Med 2013;46(1):81–95. doi: 10.1007/s12160-013-9486-6 23512568

[3] Carey RN, Connell LE, Johnston M, Rothman AJ, de Bruin M, Kelly MP, et al. Behavior change techniques and their mechanisms of action: A synthesis of links described in published intervention literature. Ann Behav Med 2019;53(8):693–707. doi: 10.1093/abm/kay078 30304386 PMC6636886

[4] Abraham C, Michie S. A taxonomy of behavior change techniques used in interventions. Health Psychol 2008;27(3):379–87. doi: 10.1037/0278-6133.27.3.379 18624603

[5] Michie S, Wood CE, Johnston M, Abraham C, Francis JJ, Hardeman W. Behaviour change techniques: the development and evaluation of a taxonomic method for reporting and describing behaviour change interventions (a suite of five studies involving consensus methods, randomised controlled trials and analysis of qualitative data). Health Technol Assess 2015;19(99):1–188. doi: 10.3310/hta19990 26616119 PMC4781650

[6] Vilardaga R, Casellas-Pujol E, McClernon JF, Garrison KA. Mobile applications for the treatment of Tobacco use and dependence. Curr Addict Rep 2019;6(2):86–97. doi: 10.1007/s40429-019-00248-0 32010548 PMC6994183

[7] Bold KW, Garrison KA, DeLucia A, Horvath M, Nguyen M, Camacho E, et al. Smartphone apps for smoking cessation: Systematic framework for app review and analysis. J Med Internet Res 2023;25:e45183. doi: 10.2196/45183 37440305 PMC10375280

[8] Orji R, Moffatt K. Persuasive technology for health and wellness: State-of-the-art and emerging trends. Health Informatics J 2018;24(1):66–91. doi: 10.1177/1460458216650979 27245673

[9] Patel ML, Hopkins CM, Brooks TL, Bennett GG. Comparing self-monitoring strategies for weight loss in a smartphone app: Randomized controlled trial. JMIR Mhealth Uhealth 2019;7(2):e12209. doi: 10.2196/12209 30816851 PMC6416539

[10] McClure EA, Tomko RL, Carpenter MJ, Treiber FA, Gray KM. Acceptability and compliance with a remote monitoring system to track smoking and abstinence among young smokers. Am J Drug Alcohol Abuse 2018;44(5):561–570. doi: 10.1080/00952990.2018.1467431 29737885 PMC6059983

[11] Yfantidou S, Sermpezis P, Vakali A. 14 years of self-tracking technology for mHealth—Literature review: Lessons learned and the PAST SELF framework. ACM Trans Comput Healthcare 2023;4(3):Article 17. doi: 10.1145/3592621

[12] Zhao J, Freeman B, Li M. Can mobile phone apps influence people’s health behavior change? An evidence review. J Med Internet Res 2016;18(11):e287. doi: 10.2196/jmir.5692 27806926 PMC5295827

[13] Wilde MH, Garvin S. A concept analysis of self-monitoring. J Adv Nurs 2007;57(3):339–50. doi: 10.1111/j.1365-2648.2006.04089.x 17233653

[14] Nahum-Shani I, Hekler EB, Spruijt-Metz D. Building health behavior models to guide the development of just-in-time adaptive interventions: A pragmatic framework. Health Psychol 2015;34s(0):1209–19. doi: 10.1037/hea0000306 26651462 PMC4732268

[15] Bandura A. Social cognitive theory of self-regulation. Organizational Behavior and Human Decision Processes 1991;50(2):248–287. doi: 10.1016/0749-5978(91)90022-L

[16] Michie S, Hyder N, Walia A, West R. Development of a taxonomy of behaviour change techniques used in individual behavioural support for smoking cessation. Addict Behav 2011;36(4):315–9. doi: 10.1016/j.addbeh.2010.11.016 21215528

[17] Barroso-Hurtado M, Suárez-Castro D, Martínez-Vispo C, Becoña E, López-Durán A. Smoking cessation apps: A systematic review of format, outcomes, and features. Int J Environ Res Public Health 2021;18(21). doi: 10.3390/ijerph182111664 34770178 PMC8583115

[18] Abroms LC, Padmanabhan N, Thaweethai L, Phillips T. iPhone apps for smoking cessation: A content analysis. Am J Prev Med 2011;40(3):279–85. doi: 10.1016/j.amepre.2010.10.032 21335258 PMC3395318

[19] Abroms LC, Lee Westmaas J, Bontemps-Jones J, Ramani R, Mellerson J. A content analysis of popular smartphone apps for smoking cessation. Am J Prev Med 2013;45(6):732–6. doi: 10.1016/j.amepre.2013.07.008 24237915 PMC3836190

[20] Kreuter MW, Farrell DW, Olevitch LR, Brennan LK. Tailoring Health Messages: Customizing Communication With Computer Technology. 1^st^ ed. New York: Routledge 2013.

[21] Kreuter MW, Skinner CS. Tailoring: What’s in a name? Health Educ Res 2000;15(1):1–4. doi: 10.1093/her/15.1.1 10788196

[22] Broekhuizen K, Kroeze W, van Poppel MN, Oenema A, Brug J. A systematic review of randomized controlled trials on the effectiveness of computer-tailored physical activity and dietary behavior promotion programs: An update. Ann Behav Med 2012;44(2):259–86. doi: 10.1007/s12160-012-9384-3 22767052 PMC3442159

[23] Lustria ML, Noar SM, Cortese J, Van Stee SK, Glueckauf RL, Lee J. A meta-analysis of web-delivered tailored health behavior change interventions. J Health Commun 2013;18(9):1039–69. doi: 10.1080/10810730.2013.768727 23750972

[24] Hébert ET, Stevens EM, Frank SG, Kendzor DE, Wetter DW, Zvolensky MJ, et al. An ecological momentary intervention for smoking cessation: The associations of just-in-time, tailored messages with lapse risk factors. Addict Behav 2018;78:30–35. doi: 10.1016/j.addbeh.2017.10.026 29121530 PMC5783727

[25] Noar SM, Benac CN, Harris MS. Does tailoring matter? Meta-analytic review of tailored print health behavior change interventions. Psychol Bull 2007;133(4):673–93. doi: 10.1037/0033-2909.133.4.673 17592961

[26] Hawkins RP, Kreuter M, Resnicow K, Fishbein M, Dijkstra A. Understanding tailoring in communicating about health. Health Educ Res 2008;23(3):454–66. doi: 10.1093/her/cyn004 18349033 PMC3171505

[27] Heffner JL, Vilardaga R, Mercer LD, Kientz JA, Bricker JB. Feature-level analysis of a novel smartphone application for smoking cessation. Am J Drug Alcohol Abuse 2015;41(1):68–73. doi: 10.3109/00952990.2014.977486 25397860 PMC4410684

[28] Battalio SL, Conroy DE, Dempsey W, Liao P, Menictas M, Murphy S, et al. Sense2Stop: A micro-randomized trial using wearable sensors to optimize a just-in-time-adaptive stress management intervention for smoking relapse prevention. Contemp Clin Trials 2021;109:106534. doi: 10.1016/j.cct.2021.106534 34375749 PMC8556307

[29] Bettinghaus EP. Health promotion and the knowledge-attitude-behavior continuum. Prev Med 1986;15(5):475–491. doi: 10.1016/0091-7435(86)90025-3 3774779

[30] Clinical Practice Guideline Treating Tobacco Use and Dependence 2008 Update Panel, Liaisons, and Staff. A clinical practice guideline for treating tobacco use and dependence: 2008 update. A U.S. Public Health Service report. Am J Prev Med 2008;35(2):158–76. doi: 10.1016/j.amepre.2008.04.009 18617085 PMC4465757

[31] Seo S, Cho SI, Yoon W, Lee CM. Classification of smoking cessation apps: Quality review and content analysis. JMIR Mhealth Uhealth 2022;10(2):e17268. doi: 10.2196/17268 35175213 PMC8895289

[32] Zhang M, Wolters M, O’Connor S, Wang Y, Doi L. Smokers’ user experience of smoking cessation apps: A systematic review. Int J Med Inform 2023;175:105069. doi: 10.1016/j.ijmedinf.2023.105069 37084673

[33] McClure JB, Hartzler AL, Catz SL. Design considerations for smoking cessation apps: Feedback from nicotine dependence treatment providers and smokers. JMIR Mhealth Uhealth 2016;4(1):e17. doi: 10.2196/mhealth.5181 26872940 PMC4769359

[34] Carroll JK, Moorhead A, Bond R, LeBlanc WG, Petrella RJ, Fiscella K. Who uses mobile phone health apps and does use matter? A secondary data analytics approach. J Med Internet Res 2017;19(4):e125. doi: 10.2196/jmir.5604 28428170 PMC5415654

[35] Régnier F, Chauvel L. Digital inequalities in the use of self-tracking diet and fitness apps: Interview study on the influence of social, economic, and cultural factors. JMIR Mhealth Uhealth 2018;6(4):e101. doi: 10.2196/mhealth.9189 29678807 PMC5935808

[36] Krebs P, Duncan DT. Health app use among US mobile phone owners: A national survey. JMIR Mhealth Uhealth 2015;3(4):e101. doi: 10.2196/mhealth.4924 26537656 PMC4704953

[37] Michie S, Jochelson K, Markham WA, Bridle C. Low-income groups and behaviour change interventions: a review of intervention content, effectiveness and theoretical frameworks. J Epidemiol Community Health 2009;63(8):610–22. doi: 10.1136/jech.2008.078725 19386612

[38] Van Rhoon L, Byrne M, Morrissey E, Murphy J, McSharry J. A systematic review of the behaviour change techniques and digital features in technology-driven type 2 diabetes prevention interventions. Digit Health 2020;6:2055207620914427. doi: 10.1177/2055207620914427 32269830 PMC7093696

[39] Iribarren SJ, Akande TO, Kamp KJ, Barry D, Kader YG, Suelzer E. Effectiveness of mobile apps to promote health and manage disease: systematic review and meta-analysis of randomized controlled trials. JMIR Mhealth Uhealth 2021;9(1):e21563. doi: 10.2196/21563 33427672 PMC7834932

[40] Wei Y, Zheng P, Deng H, Wang X, Li X, Fu H. Design features for improving mobile health intervention user engagement: Systematic review and thematic analysis. J Med Internet Res 2020;22(12):e21687. doi: 10.2196/21687 33295292 PMC7758171

[41] Diehl C, Martins A, Almeida A, Silva T, Ribeiro Ó, Santinha G, et al. Defining recommendations to guide user interface design: Multimethod approach. JMIR Hum Factors 2022;9(3):e37894. doi: 10.2196/37894 36178714 PMC9568819

[42] Sarkar U, Gourley GI, Lyles CR, Tieu L, Clarity C, Newmark L, et al. Usability of commercially available mobile applications for diverse patients. J Gen Intern Med 2016;31(12):1417–1426. doi: 10.1007/s11606-016-3771-6 27418347 PMC5130945

[43] Garrett B, Martell BN, Caraballo RS, King BA. Socioeconomic differences in cigarette smoking among sociodemographic groups. Prev Chronic Dis 2019 Jun 13;16:E74. doi: 10.5888/pcd16.180553 31198164 PMC6583815

[44] Twyman L, Bonevski B, Paul C, Bryant J. Perceived barriers to smoking cessation in selected vulnerable groups: A systematic review of the qualitative and quantitative literature. BMJ Open 2014;4(12):e006414. doi: 10.1136/bmjopen-2014-006414 25534212 PMC4275698

[45] Boland VC, Mattick RP, McRobbie H, Siahpush M, Courtney RJ. “I’m not strong enough; I’m not good enough. I can’t do this, I’m failing”: a qualitative study of low-socioeconomic status smokers’ experiences with accessing cessation support and the role for alternative technology-based support. Int J Equity Health 2017;16(1):196. doi: 10.1186/s12939-017-0689-5 29132364 PMC5683575

[46] Grimes LM, Garg R, Weng O, Wolff JM, McQueen A, Carpenter KM, et al. Appeal of Tobacco quitline services among low-income smokers. Prev Chronic Dis 2023;20:E11. doi: 10.5888/pcd20.220214 36862604 PMC9983599

[47] Businelle MS, Ma P, Kendzor DE, Frank SG, Vidrine DJ, Wetter DW. An ecological momentary intervention for smoking cessation: Evaluation of feasibility and effectiveness. J Med Internet Res 2016;18(12):e321. doi: 10.2196/jmir.6058 27956375 PMC5187451

[48] Boland VC, Stockings EA, Mattick RP, McRobbie H, Brown J, Courtney RJ. The methodological quality and effectiveness of technology-based smoking cessation interventions for disadvantaged groups: A systematic review and meta-analysis. Nicotine Tob Res 2018;20(3):276–285. doi: 10.1093/ntr/ntw391 28034998

[49] Rajani NB, Weth D, Mastellos N, Filippidis FT. Adherence of popular smoking cessation mobile applications to evidence-based guidelines. BMC Public Health 2019;19(1):743. doi: 10.1186/s12889-019-7084-7 31196062 PMC6567534

[50] Hoeppner BB, Hoeppner SS, Seaboyer L, Schick MR, Wu GW, Bergman BG, et al. How smart are smartphone apps for smoking cessation? A content analysis. Nicotine Tob Res 2016;18(5):1025–31. doi: 10.1093/ntr/ntv117 26045249 PMC5942604

[51] Hoepper BB, Siegel KR, Carlon HA, Kahler CW, Park ER, Taylor ST, et al. Feature-level analysis of a smoking cessation smartphone app based on a positive psychology approach: Prospective observational study. JMIR Form Res 2022;6(7):e38234. doi: 10.2196/38234 35900835 PMC9377446

[52] Venkatesh V, Morris MG, Davis GB, Davis FD. User acceptance of information technology: Toward a unified view. MIS Quarterly 2003;27(3):425–478. doi: 10.2307/30036540

[53] Collins LM, Baker TB, Mermelstein RJ, Piper ME, Jorenby DE, Smith SS, et al. The multiphase optimization strategy for engineering effective tobacco use interventions. Ann Behav Med 2011;41(2):208–26. doi: 10.1007/s12160-010-9253-x 21132416 PMC3053423

[54] Landoll RR, Vargas SE, Samardzic KB, Clark MF, Guastaferro K. The preparation phase in the multiphase optimization strategy (MOST): A systematic review and introduction of a reporting checklist. Transl Behav Med 2022;12(2):291–303. doi: 10.1093/tbm/ibab146 34850214

[55] National Cancer Institute Smokefree.gov. [cited 16 September 2024]. Available from: https://smokefree.gov/.

[56] Prutzman YM, Wiseman KP, Grady MA, Budenz A, Grenen EG, Vercammen LK, et al. Using digital technologies to reach tobacco users who want to quit: Evidence from the national cancer institute’s Smokefree.gov initiative. Am J Prev Med 2021;60(3 Suppl 2):S172–S184. doi: 10.1016/j.amepre.2020.08.008 33663705

[57] Bricker JB, Watson NL, Mull KE, Sullivan BM, Heffner JL. Efficacy of smartphone applications for smoking cessation: A randomized clinical trial. JAMA Intern Med 2020;180(11):1472–1480. doi: 10.1001/jamainternmed.2020.4055 32955554 PMC7506605

[58] Thomson B, Emberson J, Lacey B, Lewington S, Peto R, Islami F. Association of smoking initiation and cessation across the life course and cancer mortality: Prospective study of 410 000 US adults. JAMA Oncol 2021;7(12):1901–1903. doi: 10.1001/jamaoncol.2021.4949 34673892 PMC8532035

[59] Thomson B, Emberson J, Lacey B, Lewington S, Peto R, Jemal A, et al. Association between smoking, smoking cessation, and mortality by race, ethnicity, and sex among US adults. JAMA Netw Open 2022;5(10):e2231480. doi: 10.1001/jamanetworkopen.2022.31480 36279139 PMC9593233

[60] Doll R, Peto R, Boreham J, Sutherland I. Mortality in relation to smoking: 50 years’ observations on male British doctors. BMJ 2004;328(7455):1519. doi: 10.1136/bmj.38142.554479.AE 15213107 PMC437139

[61] Galobardes B, Shaw M, Lawlor DA, Lynch JW, Davey Smith G. Indicators of socioeconomic position (part 1). J Epidemiol Community Health 2006;60(1):7–12. doi: 10.1136/jech.2004.023531 16361448 PMC2465546

[62] Adler NE, Newman K. Socioeconomic disparities in health: Pathways and policies. Health Aff (Millwood) 2002;21(2):60–76. doi: 10.1377/hlthaff.21.2.60 11900187

[63] Shavers VL. Measurement of socioeconomic status in health disparities research. J Natl Med Assoc 2007;99(9):1013–23. 17913111 PMC2575866

[64] Wakeman M, Tesfaye L, Gregory T, Leahy E, Kendrick B, El-Toukhy S. Perceptions of mobile technologies use for smoking cessation: A focus group study with individuals of low socioeconomic status who smoke. JMIR Form Res 2024;8:e58221. doi: 10.2196/58221 39392684 PMC11512139

[65] Bispo Júnior JP. Social desirability bias in qualitative health research. Rev Saude Publica 2022;56:101. doi: 10.11606/s1518-8787.2022056004164 36515303 PMC9749714

[66] Tong A, Sainsbury P, Craig J. Consolidated criteria for reporting qualitative research (COREQ): A 32-item checklist for interviews and focus groups. Int J Qual Health Care 2007;19(6):349–357. doi: 10.1093/intqhc/mzm042 17872937

[67] Venkatesh V, Thong JYL, Xu X. Consumer Acceptance and use of information technology: Extending the unified theory of acceptance and use of technology. MIS Quarterly 2012;36(1):157–178. doi: 10.2307/41410412

[68] Braun V, Clarke V. Thematic analysis. In Cooper H., Camic P. M., Long D. L., Panter A. T., Rindskopf D., & Sher K. J. (Eds.), APA handbook of research methods in psychology, Vol 2: Research designs: Quantitative, qualitative, neuropsychological, and biological. Washington, DC, US: American Psychological Association; 2012:57–71.

[69] Friese S. ATLAS.ti 8 Windows User Manual Berlin: ATLAS.ti Scientific Software Development GmbH, 2020.

[70] Davis FD. Perceived usefulness, perceived ease of use, and user acceptance of information technology. MIS Quarterly 1989;13(3):319–40. doi: 10.2307/249008

[71] Lazard AJ, Babwah Brennen JS, Belina SP. App designs and interactive features to increase mHealth adoption: User expectation survey and experiment. JMIR Mhealth Uhealth 2021;9(11):e29815. doi: 10.2196/29815 34734829 PMC8603164

[72] Vaghefi I, Tulu B. The continued use of mobile health apps: Insights from a longitudinal study. JMIR Mhealth Uhealth 2019;7(8):e12983. doi: 10.2196/12983 31469081 PMC6740166

[73] Donevant SB, Estrada RD, Culley JM, Habing B, Adams SA. Exploring app features with outcomes in mHealth studies involving chronic respiratory diseases, diabetes, and hypertension: a targeted exploration of the literature. J Am Med Inform Assoc 2018;25(10):1407–1418. doi: 10.1093/jamia/ocy104 30137383 PMC6188510

[74] Hui CY, Walton R, McKinstry B, Jackson T, Parker R, Pinnock H. The use of mobile applications to support self-management for people with asthma: A systematic review of controlled studies to identify features associated with clinical effectiveness and adherence. J Am Med Inform Assoc 2017;24(3):619–632. doi: 10.1093/jamia/ocw143 27694279 PMC7651908

[75] Hengst TM, Lechner L, Dohmen D, Bolman CA. The facilitators and barriers of mHealth adoption and use among people with a low socio-economic position: A scoping review. Digit Health 2023;9:20552076231198702. doi: 10.1177/20552076231198702 37691766 PMC10483984

[76] Nahum-Shani I, Smith SN, Spring BJ, Collins LM, Witkiewitz K, Tewari A, et al. Just-in-Time Adaptive Interventions (JITAIs) in mobile health: Key components and design principles for ongoing health behavior support. Ann Behav Med 2018;52(6):446–462. doi: 10.1007/s12160-016-9830-8 27663578 PMC5364076

[77] Orji R, Lomotey R, Oyibo K, Orji F, Blustein J, Shahid S. Tracking feels oppressive and ’punishy’: Exploring the costs and benefits of self-monitoring for health and wellness. Digit Health 2018;4:2055207618797554. doi: 10.1177/2055207618797554 30202544 PMC6122239

[78] Thrul J, Bühler A, Ferguson SG. An Internet-based ecological momentary assessment study relying on participants’ own mobile phones: Insights from a study with young adult smokers. Eur Addict Res 2015;21(1):1–5. doi: 10.1159/000363231 25342514

[79] Tong HL, Quiroz JC, Kocaballi AB, Fat SCM, Dao KP, Gehringer H, et al. Personalized mobile technologies for lifestyle behavior change: A systematic review, meta-analysis, and meta-regression. Prev Med 2021;148:106532. doi: 10.1016/j.ypmed.2021.106532 33774008

[80] International Organization for Standardization. Ergonomics of human-system interaction: Part 210: Human-centred design for interactive systems. 2019 [cited 16 September 2024]. Available from: https://www.iso.org/standard/77520.html.

[81] Pew Research Center. Mobile Fact Sheet. 2024 [cited 28 March 2024]. In: Pew Research Center [Internet]. Available from: https://www.pewresearch.org/internet/fact-sheet/mobile/.

[82] Ceci L. Number of apps available in leading app stores 2024. 2024 [cited 16 September 2024]. In: Statista [Internet]. Available from: https://www.statista.com/statistics/276623/number-of-apps-available-in-leading-app-stores/.

[83] Ratcliff CL, Krakow M, Greenberg-Worisek A, Hesse BW. Digital health engagement in the US Population: Insights from the 2018 health information national trends survey. Am J Public Health 2021;111(7):1348–1351. doi: 10.2105/AJPH.2021.306282 34014759 PMC8493135

[84] Claudel SE, Ceasar JN, Andrews MR, El-Toukhy S, Farmer N, Middleton KR, et al. Time to listen: A mixed-method study examining community-based views of mobile technology for interventions to promote physical activity. BMJ Health Care Inform 2020;27(3):e100140. doi: 10.1136/bmjhci-2020-100140 32830106 PMC7445338

[85] Bitrián P, Buil I, Catalán S. Enhancing user engagement: The role of gamification in mobile apps. J Bus Res 2021;132:170–185. doi: 10.1016/j.jbusres.2021.04.028

[86] Nahum-Shani I, Rabbi M, Yap J, Philyaw-Kotov ML, Klasnja P, Bonar EE, et al. Translating strategies for promoting engagement in mobile health: A proof-of-concept microrandomized trial. Health Psychol 2021;40(12):974–987. doi: 10.1037/hea0001101 34735165 PMC8738098

[87] Mustafa AS, Ali N, Dhillon JS, Alkawsi G, Baashar Y. User engagement and abandonment of mHealth: A cross-sectional survey. Healthcare (Basel) 2022;10(2):221. doi: 10.3390/healthcare10020221 35206837 PMC8872344

[88] Presnsky M. Digital natives, digital immigrants. On the Horizon 2001;9(5).

