# Correction: A feature-based qualitative assessment of smoking cessation mobile applications

**DOI:** 10.1371/journal.pdig.0001307

**Published:** 2026-03-19

**Authors:** Lydia Tesfaye, Michael Wakeman, Gunnar Baskin, Greg Gruse, Tim Gregory, Erin Leahy, Brandon Kendrick, Sherine El-Toukhy

S1–S7 Tables were uploaded incorrectly. Please see the correct S1–S7 Tables here.

## Supporting information

S1 Note Moderation guide. (DOCX)

S2 Note Perceptions of apps’ names and landing pages. (DOCX)

S1 Table Consolidated criteria for reporting qualitative research (COREQ). (DOCX)

S2 Table Detailed participant characteristics. (DOCX)

S3 Table Themes and illustrative quotes of individuals who smoke on the self-monitoring feature in QuitGuide and Quit Journey. (DOCX)

S4 Table Themes and illustrative quotes of individuals who smoke on the tailored feedback and support feature in QuitGuide and Quit Journey. (DOCX)

S5 Table Themes and illustrative quotes of individuals who smoke on the educational content feature in QuitGuide and Quit Journey. (DOCX)

S6 Table Themes and illustrative quotes of overall perceptions of QuitGuide and Quit Journey, names, and landing pages. (DOCX)

S7 Table Illustrative quotes of suggestions to improve QuitGuide and Quit Journey. (DOCX)
