# Supplementary material for: A feature-based qualitative assessment of smoking cessation mobile applications
Source: PLOS Digit Health. 2024 Nov 21;3(11):e0000658. doi: 10.1371/journal.pdig.0000658 (PMC11581403; doi:10.1371/journal.pdig.0000658)
Supplement: S1 Note — (DOCX) [file pdig.0000658.s001.docx]

**S1 Note. Moderation guide.**

Introduction

I’d like to hear your thoughts about a smoking cessation mobile phone app that the sponsors of this focus group have developed.

1. What do you think of the app name? What do you like/dislike about it? If you were to change the name, what would it be?
2. What do you think of the app’s landing page? What do you like/dislike about it? What do you suggest to improve it?

Main topics

The app allows users to:

(a) set a quit date.

(b) track whether they have stayed smoke-free or have slipped, their cravings and mood.

(c) personalize their quit plan whereby users can, for example, list their own reasons for quitting or decide what to do after recording a slip or craving (e.g., requesting help at this time or location in the future).

(d) visualize their smoking behavior and progress (e.g., calendar view of slipping entries, quit journey view of quitting milestones).

(e) access to information about how to quit and other quit resources.

For each of the above features, we asked participants:

1. What do you like/dislike about the [feature]? What do you suggest to improve it?
2. How useful would an app with this feature be to you?
3. Do you think this would be fun to use?
4. Do you think this would be easy to use?
5. (Post design only) What do you think of the app’s screens we showed you?

Concluding Questions

1. What is your overall impression of the app?
2. Of the features we presented, what features would be most useful to you?
3. What additional features would you prefer the app have?

Note: Questions were near identical in all focus group discussions. In focus groups 1 through 8, participants were shown screenshots from QuitGuide while participants in focus groups 9 through 12 were shown screenshots from Quit Journey.
