# Supplementary material for: A feature-based qualitative assessment of smoking cessation mobile applications
Source: PLOS Digit Health. 2024 Nov 21;3(11):e0000658. doi: 10.1371/journal.pdig.0000658 (PMC11581403; doi:10.1371/journal.pdig.0000658)
Supplement: S2 Note — (DOCX) [file pdig.0000658.s002.docx]

**S2 Note. Perceptions of apps’ names and landing pages.**

A total of 39 quotes pertained to the apps’ names, Quit Guide and Quit Journey (*n*= 25 and *n*= 14, respectively). Participants had negative opinions about the names of both apps. Although some voiced liking that “Quit” was part of the names, the names were not specific to smoking cessation, which made it harder to identify them as such while browsing the App store or Google Play.

*I like that quit is in the name, but I think that with Quit Journey, it's not very clear that it's about smoking specifically. So if I was to see it in the app store, I wouldn't necessarily be drawn to it because I wouldn't really be sure … what it is about. (P29, QJ)*

*I think it should be something simple with smoking in the name … It has to be something I can associate with that right off the bat, otherwise I'll forget about it. (P28, QJ)*

Furthermore, the word “journey” generated mixed opinions where some participants indicated the word reflects the reality of quitting whereas others indicated it implied a long process.

*… the whole word journey sounds like it's going to be a tedious process, like it's going to be a frustrating one that takes way too long. (P28, QJ)*

*I think that the word journey kind of indicates that like most of us who've tried quitting or have quit, we understand that it's not a quick process and … the name … entails it's a journey and … it's a long one sometimes for most of us. (P14, QJ)*

Reactions to the landing pages of both apps were mixed. Regarding the color scheme, many participants remarked that the color scheme of QuitGuide was dark, whereas they appreciated Quit Journey’s bright colors. Participants thought that the landing pages of both apps were simple and easy to navigate.

*I was thinking maybe brighter colors [on the landing page], the colors are like a little dark and some people, you know, they smoke because of depression. So, … when I think of dark colors, I think, you know, depressing … So, maybe like more brighter colors or, you know, just more of a … feel-good background. (P16, QG)*

*I think [the landing page] looks pretty stylish. I like the colors a lot. (P10, QJ)*

*Yeah, I like [the landing page] … It's streamlined and simple. I can already tell what all of the options to click on there do. (P05, QG)*

Participants were generally willing to try or intending to use both apps, especially if endorsed by someone in their social circles.

*I would love to download [the app]. Also, I think that every day that I do smoke a cigarette I always think about ways to quit smoking. So, I would love to try it. (P25, QJ)*

*If I saw someone using [the app] and that it was doing well for them, then I would want to try. (P10, QG)*
