# Supplementary material for: A feature-based qualitative assessment of smoking cessation mobile applications
Source: PLOS Digit Health. 2024 Nov 21;3(11):e0000658. doi: 10.1371/journal.pdig.0000658 (PMC11581403; doi:10.1371/journal.pdig.0000658)
Supplement: S1 Table — (DOCX) [file pdig.0000658.s003.docx]

**S1 Table. Consolidated criteria for reporting qualitative research (COREQ).**^66^

| Topic | Guide Questions/Description | Page No. |
| --- | --- | --- |
|  |  |  |
| Domain 1: Research team and reflexivity |  | 1 |
|  |  |  |
| *Personal Characteristics* |  |  |
| Interviewer/facilitator | Which author/s conducted the interview or focus group? | 7 |
| Credentials | What were the researcher’s credentials? | 7 |
| Occupation | What was their occupation at the time of the study? | 7 |
| Gender | Was the researcher male or female? | 7 |
| Experience and training | What experience or training did the researcher have? | 7 |
|  |  |  |
| *Relationship with participants* |  |  |
| Relationships established | Was a relationship established prior to study commencement? | 7 |
| Participant knowledge of the interviewer | What did the participants know about the researcher? | 7 |
| Interviewer characteristics | What characteristics were reported about the interviewer/facilitator? | 7 |
|  |  |  |
| Domain 2: Study design |  |  |
|  |  |  |
| *Theoretical framework* |  |  |
| Methodological orientation and Theory | What methodological orientation was stated to underpin the study? | 6-8 |
|  |  |  |
| *Participant selection* |  |  |
| Sampling | How were participants selected? | 7 |
| Method of approach | How were participants approached? | 7 |
| Sample size | How many participants were in the study? | 7 |
| Non-participation | How many people refused to participate or dropped out? | 7 |
|  |  |  |
| *Setting* |  |  |
| Setting of data collection | Where was the data collected? | 7 |
| Presence of non-participants | Was anyone else present besides the participants and researchers? | 7 |
| Description of sample | What are the important characteristics of the sample? | 18 |
|  |  |  |
| *Data collection* |  |  |
| Interview guide | Were questions, prompts, guides provided by the authors? Was it pilot tested? | 7-8 |
| Repeat interviews | Were repeat interviews carried out? If yes, how many? | 8 |
| Audio/visual recording | Did the research use audio or visual recording to collect the data? | 7 |
| Field notes | Were field notes made during and/or after the interview or focus group? | 7 |
| Duration | What was the duration of the interviews or focus group? | 7 |
| Data saturation | Was data saturation discussed? | 7 |
| Transcripts returned | Were transcripts returned to participants for comment and/or correction? | 7 |
|  |  |  |
| Domain 3: Analysis and findings |  |  |
|  |  |  |
| *Data analysis* |  |  |
| Number of data coders | How many data coders coded the data? | 8 |
| Description of the coding tree | Did authors provide a description of the coding tree? | 8 |
| Derivation of themes | Were themes identified in advance or derived from the data? | 8 |
| Software | What software, if applicable, was used to manage the data? | 8 |
| Participant checking | Did participants provide feedback on the findings? | 7 |
|  |  |  |
| *Reporting* |  |  |
| Quotations presented | Were participant quotations presented to illustrate the themes/findings? Was each quotation identified? | 8-13 |
| Data and findings consistent | Was there consistency between the data presented and the findings? | 8-13 |
| Clarity of major themes | Were major themes clearly presented in the findings? | 8-13 |
| Clarity of minor themes | Is there a description of diverse cases or discussion of minor themes? | 8-13 |
|  |  |  |
