# Supplementary material for: A feature-based qualitative assessment of smoking cessation mobile applications
Source: PLOS Digit Health. 2024 Nov 21;3(11):e0000658. doi: 10.1371/journal.pdig.0000658 (PMC11581403; doi:10.1371/journal.pdig.0000658)
Supplement: S2 Table — (DOCX) [file pdig.0000658.s004.docx]

**S2 Table. Detailed participant characteristics.**^64^

| **ID** | **Sex** | **Race and ethnicity** | **Highest level of education** | **Smoking frequency** | **Quit timeframe** | **Smartphone operating system** |
| --- | --- | --- | --- | --- | --- | --- |
|  |  |  |  |  |  |  |
| P01^*^ | Female | NH White | Some college, ND | Every day | 30 days | Android |
| P02^*^ | Male | Hispanic/Latino | Some college, ND | Every day | 30 days | Android |
| P03 | Female | Hispanic/Latino | HS incomplete | Every day | 7 days | Android |
| P04^*^ | Female | NH White | Some college, ND | Some days | 30 days | Android |
| P05 | Male | NH White | Some college, ND | Every day | 7 days | Android |
| P06 | Male | NH Black/AA | Some college, ND | Every day | 30 days | Android |
| P07 | Male | NH Asian, NHPI | HS equivalent | Every day | 30 days | iOS |
| P08^*^ | Female | Hispanic/Latino | Some college, ND | Every day | 30 days | iOS |
| P09 | Male | NH White | HS equivalent | Every day | 30 days | iOS |
| P10^*^ | Male | NH White | HS graduate | Every day | 7 days | Android |
| P11^*^ | Female | Hispanic/Latino | Some college, ND | Every day | 30 days | iOS |
| P12^*^ | Male | NH White | Some college, ND | Every day | 7 days | Android |
| P13^*^ | Female | NH White | HS graduate | Every day | 30 days | Android |
| P14^*^ | Female | NH White | Two-year AD | Some days | 30 days | iOS |
| P15^*^ | Female | NH Black/AA | HS incomplete | Some days | 30 days | Android |
| P16^*^ | Female | AIAN | HS incomplete | Every day | 30 days | iOS |
| P17^*^ | Male | NH Black/AA | HS graduate | Every day | 30 days | Android |
| P18 | Female | NH Black/AA | Some college, ND | Every day | 7 days | iOS |
| P19 | Female | NH White | HS equivalent | Every day | 7 days | Android |
| P20 | Female | NH White | HS graduate | Every day | 7 days | Android |
| P21 | Female | NH White | Some college, ND | Every day | 7 days | iOS |
| P22 | Female | NH White | Some college, ND | Every day | 30 days | iOS |
| P23 | Male | NH White | Some college, ND | Every day | 7 days | iOS |
| P24 | Female | NH Black/AA | Some college, ND | Every day | 30 days | Android |
| P25 | Female | NH Black/AA | HS graduate | Some days | 30 days | Android |
| P26 | Male | NH Black/AA | Some college, ND | Every day | 30 days | iOS |
| P27 | Male | Hispanic/Latino | Some college, ND | Some days | 30 days | Android |
| P28 | Female | NH White | Two-year AD | Every day | 30 days | Android |
| P29 | Female | NH White | Some college, ND | Every day | 30 days | iOS |
| P30 | Male | NH Asian, NHPI | HS graduate | Some days | 6 months | Android |
| P31 | Male | NH Black/AA | Some college, ND | Every day | 6 months | Android |
| P32 | Female | NH Asian, NHPI | HS graduate | Every day | 30 days | iOS |
| P33 | Male | Hispanic/Latino | Two-year AD | Some days | 6 months | iOS |
| P34 | Male | NH Black/AA | Two-year AD | Every day | 6 months | Android |
| P35 | Female | NH Black/AA | HS graduate | Every day | 30 days | iOS |
| P36 | Male | NH White | Some college, ND | Every day | 7 days | Android |
| P37 | Male | NH Black/AA | HS graduate | Every day | 6 months | iOS |
| P38 | Male | NH Mixed | HS graduate | Some days | 7 days | iOS |

NH = Non-Hispanic, AA= African American, NHPI = Native Hawaiian/Pacific Islander, AIAN= American Indian, Alaska Native
HS = high school, ND = no degree, AD = associate degree

Participants who identified as Hispanics or Latinos were considered as such regardless of race.

^*^Participated in two focus groups.
