# Supplementary material for: A feature-based qualitative assessment of smoking cessation mobile applications
Source: PLOS Digit Health. 2024 Nov 21;3(11):e0000658. doi: 10.1371/journal.pdig.0000658 (PMC11581403; doi:10.1371/journal.pdig.0000658)
Supplement: S3 Table — (DOCX) [file pdig.0000658.s005.docx]

**S3 Table. Themes and illustrative quotes of individuals who smoke on the self-monitoring feature in QuitGuide and Quit Journey**

| **Themes** | **App** | **Quotations** | **Sentiment** |
| --- | --- | --- | --- |
| Performance Expectancy | QG | P22: I do think it would be helpful just to see where you screwed up, then you can see what your triggers were maybe for your next quit round. | Positive |
|  | QG | P24: I think [tracking is] kind of helpful. It gives you … some type of accountability. You wouldn't really want to download the app if you don't think you're going to use it … It's giving you options other than just pressing a button and saying I didn't smoke today. | Positive |
|  | QG | P24: Seeing … if you have … good days, you started to see that okay, it’s possible, and let me just keep up with myself.^1^ | Positive |
|  | QG | P22: I think [tracking is] useful … It'd be interesting if you can … look back at the data to see … I'm in the car and that's when I usually crave cigarettes, maybe I won’t … bring them back with me to the store or something. [It makes you] a little more aware. | Positive |
|  | QG | P23: I think that's nice, because it shows you … exactly … what you're slipping on and … what you need to work on. So, I feel like it's kinda nice in that way.^1^ | Positive |
|  | QG | P24: I feel [tracking] makes you more serious about what you're doing because there's a lot of data and it's right in front of you. You don’t have to hope and see the results in the mirror. You can see the results on the bar graph and stuff.^1^ | Positive |
|  | QG | P22: I think that [tracking is] a useful part of the app, definitely. So, when you have a day where you mess up, though it doesn't start over, it's still marking your data down. So, I like that part of it.^1^ | Positive |
|  | QG | P13: [I] like … the track my craving part because … that’s a big issue … Even like trying to quit, it’s just like the cravings and stuff. So, maybe it can help … with that, so that’d be kinda cool. | Positive |
|  | QG | P13: To me, [the calendar option] just helps me … it would help me keep track a lot more.^1^ | Positive |
|  | QG | P11: I think [tracking], that’s super cool … I think … being just more aware and mindful of … why you might have smoked a cigarette always helps you to be more mindful and then … makes quitting a lot easier. | Positive |
|  | QG | P13: I think it would be pretty good … because … keeping track of … what went wrong and … why you went back to smoking or … What is making you smoke is … hard to remember and then you know [you’re] kind of just like, okay, whatever I'm just gonna keep doing it, and then I'll quit later or something, so an app like this would help a lot. Just keeping track of everything. | Positive |
|  | QG | P12: [Tracking] could be super, super useful because … the first thing with your habits is … you have to understand … what they are … oh, I'm always smoking in the morning or the evening … If it's tailored to the individual then that could be super helpful in identifying … where you're going wrong and where you need the most help correcting.^1^ | Positive |
|  | QG | P05: To me, it would be really useful just because I have a pretty busy day-to-day, so being able to track would help me kind of keep tabs on it. Otherwise, I may, you know, forget. | Positive |
|  | QG | P06: I think the [tracking] feature would allow me to predict my future movements and my future cravings so that I can maybe try to avoid the triggers for my cravings. | Positive |
|  | QG | P06: Yeah, [tracking] would be really useful. | Positive |
|  | QG | P04: It would be cool just to see how well you're doing or how bad you're doing versus just kind of winging it by ear or … not keeping track of it.^1^ | Positive |
|  | QG | P20: I think [tracking is] a wonderful thing … I’ve struggled with addiction in my life before. So, I think it's very, very important to … recognize what triggers are, where … they come from, and where they start … I think it’s a positive thing and an important thing to have on an app. | Positive |
|  | QG | P21: I think one of the most important things with trying to quit smoking is like tracking when you smoke the most, and what kind of triggers cause you to smoke, like is it eating or stress … or things like that. So, I think just having an easy way to see that would make you … be able to avoid certain triggers to cut down on the cigarettes themselves. | Positive |
|  | QG | P21: I really like the idea of the trigger thing … That's probably the most important part in quitting smoking, would be to identify your triggers, like, for me, having a cup of coffee in the morning. It's to the point where I always like associate having my morning cup of coffee with having a cigarette … it probably goes into, like, behavior modification … That's one that I can identify right away, but I'm sure that there's a lot more that I don't necessarily identify that are more habits of when I do smoke, or things that happen, that causes me to smoke. | Positive |
|  | QG | P20: I think [tracking is] a wonderful thing … I think it's a main tool that you have to have in order to not just … quit smoking or quit anything but stay that way. | Positive |
|  | QG | P16: I like that … when you clicked on “I slipped” … there was a message that … gave some motivation … Some people don't have a lot of support and … it was nice to see that because … people that are trying to stop smoking, it's really, really hard and that comes from a person that has been smoking for a few years now and I've tried several times, so I thought that was really good. | Positive |
|  | QG | P16: I like how the days that you went without smoking [are] green and then you can see the days that you slipped are just super red … You can see like okay I've been doing really great. I’ve only had like two slip ups … I see the good and the bad, so it helps me … I'm a visual learner. So, I think I like the way it's set up like that with the colors that you can see your slip ups and your good days just right there, together.^1^ | Positive |
|  | QG | P14: I like the fact that there is positive reinforcement built within the app [when someone tracks a slip] to remind the person utilizing it. | Positive |
|  | QG | P16: I think it would be helpful to others … if they can see their reasons … [and] they can go back and see their reasons for slipping … say for … a month's time. [If] I can see every time I slipped in my reason for slipping I think that will be a help. | Positive |
|  | QG | P03: Yeah, I like [tracking] too. The thing that really caught my eye was the fact that you can track your cravings … That’d probably be a key point for most smokers, I know me … for instance. | Positive |
|  | QG | P01: I think [tracking] could definitely be helpful. | Positive |
|  | QG | P02: I'm a pretty analytical person and I feel like …looking at graphs [are] also just different forms of like … positive reinforcement. It only helps me … to feel like I've accomplished … not succumbing … to having a cigarette.^1^ | Positive |
|  | QG | P03: I like that it has the "I slipped” button too … to make yourself kind of guilty. Like, man, I was doing so good … That makes sense. | Positive |
|  | QG | P01: Maybe I'll be more likely to be like, oh, well, it's not going to hurt anything … and smoke it if I wasn’t keeping track of it … I'm not good with … remembering what day I did something or what day something happened usually. It just kind of blurs together after a while. | Positive |
|  | QG | P03: Yeah, [the tracking features are] giving you accountability. | Positive |
|  | QG | P02: I like [tracking], but I also think … I'm an analytical person, so … it helps me realize … when I need to be a little more accountable and stronger. | Positive |
|  | QG | P18: So … you're saying that it’s gonna … help you with tracking your mood and your craving cause I know that has a lot to do with smoking. You know your mood … and how you’re feeling. Sometimes … after I eat like, I wanna smoke … Help [with] tracking the moods and then your cravings [so] then I think after that it’ll be like, “well how many cigarettes am I smoking when my mood changed to this”, or “when I’m craving this do I crave a cigarette also?” So I think that's kinda cool. | Positive |
|  | QG | P05: I think it would be … better than the alternative of not having a visual for it. Because as you know, a month or two goes by, you're not going to remember how you did on [each] day. So, this is a way to kinda … look back and see where you're at [and] where you were. | Positive |
|  | QG | P01: Well, [tracking] allows you to actually look at your progress to see okay, I've done this for nine days [and] don't want to mess up now. | Positive |
|  | QG | P02: I like the progress too, I think [tracking] just gives me something to fall back onto to realize how far I've come. | Positive |
|  | QG | P19: Yeah, I think [visualizing progress] would be pretty … cool … A bar graph or something showing you … like the bars go up and down just to show you what you used and like for what period of time. That way, at least you ... know how much you're smoking and you know that you weren't smoking this much … and you can see the difference.^1^ | Positive |
|  | QG | P10: I think it seems helpful to have the option to ask for help for sure [at a certain time or place after tracking a slip]. Like to know when your cravings are the worst and to be able to track them down and get an idea of when the worst times are. | Positive |
|  | QG | P08: Graphs are pretty helpful for me as well because you get to see … your history of smoking [with] how much you smoke in a day and … when you're trying to quit, how many cigarettes you tend to smoke in a day might be super helpful and beneficial to stop smoking.^1^ | Positive |
|  | QG | P09: [Tracking] gives you a broader sense of how much you're actually dying down your smoking. | Positive |
|  | QG | P10: I feel like visual options help … you get a better idea of … the timeline of quitting and the benefits of quitting. I [have] always been a fan of graphs and bar graphs … so if it has your health updates on there, that could be beneficial for me.^1^ | Positive |
|  | QG | P18: I think the graph, where you can see like the numbers and like how much you smoke and stuff, [is the thing I like best about the app].^1^ | Positive |
|  | QG | P19: My favorite thing [about the app] would probably [be] like how you can see the graphs on … how much you're really smoking to see how much of it you're really putting in your body.^1^ | Positive |
|  | QG | P24: What I really did like was … when … it would have … data showing … like bar graphs and … actual progress being shown. Cause unlike other apps Like this would actually show results from you keeping data … … you'd have to tell the truth. There's no reason to lie to yourself that you didn't smoke. You’ll know you smoked. So, yes, I like that.^1^ | Positive |
|  | QG | P23: I liked the tracking also, I think that was a pretty cool feature. I think it's nice that it shows like your progress right away.^1^ | Positive |
|  | QG | P23: I think that's … important for somebody who's like on their phone all the time and … always looking at … that app, but I feel like you could get distracted and … not look at it for a couple of hours and … smoke and then get back into it without even noticing, and you kind of forget about your app and forget … that it's being tracked and stuff. | Neutral |
|  | QG | P18: I think [visualization of progress is] useful for people that want to stop smoking … if they have a real purpose on quitting.^1^ | Neutral |
|  | QG | P07: [How useful tracking is] just kind of [depends on] where you are during the day and how many times you look at it. | Neutral |
|  | QG | P13: Yeah, I don’t understand [the visualization options] either and … if it’s like even a little bit what I think [then] I don't even think it's helpful. It's just like these are the days you’re sad kind of thing … I don't really like this.^1^ | Negative |
|  | QG | P21: I didn't like … the color. I didn't really like how red it was. I feel like … it almost feels like a failure.^1^ | Negative |
|  | QG | P13: I don't really like the [red] color [of the tracking slips background] either, it's [feels] … like you've done something wrong like bad or something. And I get that's what you did, you've like messed up but it just seems like it would probably make you feel way worse and not want to get back on track in my opinion. ^1^ | Negative |
|  | QG | P22: I feel like if … I say [that] I was not smoking for a week and I'd been pressing the … smoke free [button] all week and then I had to press I slipped and it was like … “you're starting at zero again” and there was no words of encouragement, I might just go “I’m leaving this app, it made me feel bad”. | Negative |
|  | QJ | P34: I like that … it holds you accountable … like the reason why you slipped … and like your current mood … So that way, you can track … why [you] keep slipping. | Positive |
|  | QJ | P31: I think that's a good idea. It will track your mood and why you did it, and then you can go back and look how many times you did and why you did it. | Positive |
|  | QJ | P35: I think [tracking] is a way of just knowing … what's triggering your smoking … I think it's a great way to me to be accountable for that, and just to watch out, like, hey, maybe I should stay away from this because it makes me want to smoke. So, yeah, it's a way of just knowing your triggers. | Positive |
|  | QJ | P36: I think that [tracking is] like a helpful thing for self reflection. | Positive |
|  | QJ | P04: I like how it says … like how many minutes you saved up not going out and smoking … It counts everything right there for you, which is kinda cool, and I think would give me more motivation, to be like, oh, I'm saving this much money, let me keep going. | Positive |
|  | QJ | P14: If this tracks the history, like I see at the bottom, I think that that's great … to truly understand your habits and … really see what exactly might be a reoccurring issue of what's triggering you or causing you to slip and trying to make changes on a daily basis to avoid those situations or factors and feelings that you're getting. | Positive |
|  | QJ | P04: I think that it’s useful cause I’m seeing … what kinda mood I was in that day or if I did slip [what] my reasoning was, and it could help me like put it out there and … physically see it out versus just … saying, “Oh, well, this is why this is,” versus actually seeing it on the phone or something. | Positive |
|  | QJ | P25: I also like how it says how many cigarettes you’ve avoided. Yeah, it kind of shows you your progress as you go. | Positive |
|  | QJ | P16: I think that's really helpful, because some people … they like to see it. I'm a visual learner, so I like to see my progress, and I like to see when I slipped up and stuff. So, I think that's really great to be able to track.^1^ | Positive |
|  | QJ | P25:I really like the mood [tracking] because it’s like everyday my mood is up and down, and some days I want a cigarette more than others. So I think this … mood scale is really nice and then you can also see how … you felt that day and … see what you can change as far as your mood and you know to help you not want a cigarette.^1^ | Positive |
|  | QJ | P26: I think about … the time saved, because … you do waste a lot of time just sitting there, when you could be doing something else. | Positive |
|  | QJ | P13: I think it would also just be good because … when you're trying to quit smoking … certain emotions, or maybe even where you are, or something that you've just done can … sort of trigger [you]. [To have something] tell you … this made you smoke last time or have a craving would be very helpful. | Positive |
|  | QJ | P28: It'll help you be more mindful of … every time you feel a certain way or have a certain mood that … contributes to you needing this smoke. | Positive |
|  | QJ | P29: I think it would be very useful, because … with smoking, it is a very emotional thing, especially when you're trying to quit … I've never really found anything where I can track my mood and track how much I smoke. So, I think it would be really helpful. | Positive |
|  | QJ | P13: When you're going through certain … cravings and being able to keep up with them and realize … when things are happening, it's really helpful. | Positive |
|  | QJ | P28: I think it's great that you can keep track of when you slipped or what was the reason behind it so that you can get a better … control of that. | Positive |
|  | QJ | P11: I do like how [when tracking] it says … “Let's try again”, so it's … a little motivating and not … burdening you for having a slip. | Positive |
|  | QJ | P14: I do like how [when tracking] says … “Let's try again” so it's … a little motivating … and I really like that because it's a huge factor I'm sure for most of us, especially myself. | Positive |
| Effort Expectancy | QG | P18: I think [tracking] will be pretty easy. | Positive |
|  | QG | P24: I don't think [being able to visualize progress would] be that hard, as long as you keep being honest, I guess, it'll show you the required data that could help you quit.^1^ | Positive |
|  | QG | P23: Everything kind of puts you in the right direction, you just have to follow the steps.^1^ | Positive |
|  | QG | P05: Yeah, [tracking] … looks really simple. | Positive |
|  | QG | P03: [Tracking] looks pretty simple. | Positive |
|  | QG | P05: Personally, bar graphs are a lot easier for me to understand than other people who may be the complete opposite. So, I think that … it just makes it more accessible to your average person.^1^ | Positive |
|  | QG | P05: [The visualization feature] looks pretty simple | Positive |
|  | QG | P13: [The calendar] just visually looks better to me. It's easier to keep track of things and to align it with … my other things that I do with calendars every day. So that's just why I like it.^1^ | Positive |
|  | QG | P09: Most definitely if it did everything for me, [visualizing progress would] be really user friendly.^1^ | Neutral |
|  | QG | P11: [Tracking is] easy if [the app] has reminders to do it because oftentimes I'll forget. | Neutral |
|  | QG | P02: [Tracking] looks easy. As long as it opens fine and [is] quick when I'm dealing with the craving, I don’t have a problem. | Neutral |
|  | QG | P01: [Tracking] looks fairly simple to me, as long as it's quick and I don't have to use too much time recording my cravings and things like that. | Neutral |
|  | QG | P11: I think [tracking would be easy to use] if you don't have to … do certain things in order for it to work … So like having [it be] as … hands-on or hands off as you want … would … make it easier. | Neutral |
|  | QG | P12: I didn't have a smartphone for a long time either so yes, if [the app] had … premade responses that you could just click, [tracking] would be even simpler. | Neutral |
|  | QJ | P31: [Tracking] looks really simple to use … it would be easy. | Positive |
|  | QJ | P36: [Tracking] looks simple to use for sure. | Positive |
|  | QJ | P04: [Tracking] looks really straightforward. | Positive |
|  | QJ | P14: [Tracking] seems very simple. | Positive |
|  | QJ | P12: [Tracking] looks pretty simple. | Positive |
|  | QJ | P12: I like the simplicity of [the tracking slips page].^1^ | Positive |
|  | QJ | P30:I feel like [tracking] could become … part of a daily routine or something like you wake up in the morning, use it once, go again in the evening, and use it again. Like, it doesn't look too hard to [use] … you don't really have to … go out of your way to record. So, you can pretty much do it anytime. It's something … that you can use daily. | Positive |
|  | QJ | P30: Yeah, I think … the mood is a kind of … an easy way to guess … any time of the day or whatever. You can just kind of … swipe what you're feeling. It's not like you have to go out of your way to rank or whatever. | Positive |
|  | QJ | P13: I think [tracking] would be like a routine that you could try to use.^2^ | Positive |
|  | QJ | P28: I can see [tracking] being a thing I would use a few times a day or however many times I need it. Maybe a check in the morning and a check in at night.^2^ | Positive |
|  | QJ | P13: I personally like [the tracking craving page] a lot [with] how simple it is overall.^1^ | Positive |
|  | QJ | P13: I like how simple [the tracking craving page] looks and I like … the colors and everything. It’s just plain [and] … nice.^1^ | Positive |
|  | QJ | P28: I really like … [that the tracking craving page is] simple and … basically what you see is what you get. ^1^ | Positive |
|  | QJ | P14: [The tracking slips page is] pretty bland. It just, it seems like it's almost using very old type of software system because it's so simple. But that may, for some people, be easier and more appealing … For me, I like more of a modern look.^1^ | Neutral |
|  | QJ | P12: I don't know if I would feel like typing out a reason every time I smoked. | Negative |
| Facilitating Conditions | QG | P11: There have been some apps [for quitting] alcohol where they have like the money [saved] thing and for me, that's … also inspiring to be like, wow, I've saved this much money from … not smoking this much. | Positive |
|  | QG | P07: For some really committed to wanting to stop smoking … it would vary from person to person … Sometimes when you're out for a cigarette, you're not really looking at your phone or sometimes you are, but you're watching videos, and there's so many other distractions … It’s gotta be something you have to … remind yourself every day and if it did give you notifications, that can either be … annoying to some people or helpful for the people that I guess do want to quit. | Neutral |
|  | QG | P09: If I gave [tracking a rating] out of a ten…And, if I had it in front of me and I was actually determined on using the app, then it probably [would] be an eight out of the ten. If I wasn't really all determined on quitting, … probably … really low, around two out of ten. | Neutral |
|  | QG | P08: But I do think [tracking] would be pretty helpful as far as … it kind of depends on how committed you are to smoking. | Neutral |
| Hedonic Motivation | QG | P07:When you actually are [seeing] the progress and you're doing the steps or you can see kind of physically in front of you … how much you've cut down, I think then it kind of becomes fun … especially if you're a competitive person [and] you like to see those results in front of you .. It kind of gives you confidence … So … I think it would be hard at first, because it's just something you have to get used to. | Positive |
|  | QG | P24: I think it’ll be exciting to see progress. Though, you might start off saying … oh I really had a bad day today [and] didn't know how to take care of my nerves, compared to maybe, like day three, where like oh, it feels good to talk about my cravings instead of just fulfilling them. | Positive |
|  | QG | P23: Yeah, I guess [visualization of progress] would be fun.^1^ | Positive |
|  | QG | P11: I think [tracking is fun], but I'm … kind of like [into] data [and] stuff like that. | Positive |
|  | QG | P05: I think [tracking] could be fun to see … how you improved … [and] how well you're doing. As opposed to just … kinda trying to do it on your own or in your head [and] … not keeping track at all. | Positive |
|  | QG | P04: I think I would enjoy [tracking]. | Positive |
|  | QG | P04: I think [visualizing progress would be fun], just so people can see. Because everyone … does have … different ways [that] they like to track things … So having different views on it … would be fun to just be able to see your progress.^1^ | Positive |
|  | QG | P09: I mean it really does depend on your sense of fun. | Neutral |
|  | QG | P13: I don't know if [tracking] would be fun because I've never used apps like this … It might be a little bit too overwhelming … I don't know if it's like possible to make it .. less overwhelming, but it might be fun, as well. | Neutral |
|  | QG | P05: I was just going to say that the fun factor … could depend on how interactive [tracking is] … [and] how many different ways there are to input things. | Neutral |
|  | QG | P08: I don't think [tracking] would be necessarily fun. | Neutral |
|  | QG | P10: In terms of just like tracking and being notified, that probably wouldn't be too fun for me, per se. | Negative |
|  | QG | P10: I'm not sure if [tracking] would constitute fun for me. | Negative |
|  | QG | P22: For me, I don't think [tracking] would be that fun, just because it would … put me really into my addiction and [make me] very aware of [it] … almost like too aware. | Negative |
|  | QG | P12: I can’t say that [tracking] would be fun because that's just not my idea of fun. | Negative |
|  | QG | P07: I don't think [tracking] would be fun in the sense when you first start it. | Negative |
|  | QJ | P11: [Tracking] doesn’t look particularly like fun, but it's not horrible either. It doesn't look like it's going to be like too boring and monotonous, I think the [icons] are kind of cute.^1^ | Neutral |
|  | QJ | P14: I don't know that it would necessarily [be] fun. | Neutral |
|  | QJ | P13: But if it was just sensing your mood, I wouldn’t consider that fun, more like just a routine kind of thing | Neutral |
|  | QJ | P38: This [mood tracking] screen? No, it doesn't really look too fun, but the [landing page] … looks a little more better than this one.^1^ | Neutral |
| Not applicable | QG | P23: I think I would personally use this [tracking feature] just to try it out at first and see.^2^ | Positive |
|  | QG | P11: I think it's good, people like looking at different things. Also just like seeing your data represented in different ways can be interesting.^1^ | Positive |
|  | QG | P11: My favorite is probably the line graph … Can see all the data [and] … that's interesting for me.^1^ | Positive |
|  | QG | P19: Now, [the visualization options], that's pretty cool.^1^ | Positive |
|  | QG | P17: Yeah, [calendars and charts] a good idea.^1^ | Positive |
|  | QG | P24: I think the tracking feature is a good feature, it makes it more personalized. | Positive |
|  | QG | P22: I like that part of it, that you can kinda make it for you [by tracking your cravings]. | Positive |
|  | QG | P22: I like that there's multiple options … to view [the tracking data].^1^ | Positive |
|  | QG | P13: I like the calendar. The other stuff is I don't know, it's kinda whatever to me, but … I can see why other people would really like that though. For me, though, I just really like the calendar, I'm a very calendar type of person.^1^ | Positive |
|  | QG | P13 : Overall, I like the trigger [tracking] part. | Positive |
|  | QG | P21: I think [the visualization options are] cool because … each serves its own purpose … So, like the calendar … you see it as a whole or like … the line graph, you know, you could have that more for like a week, you know.^1^ | Positive |
|  | QG | P16: I think [the visualization options are] great because I am a visual learner. So, a lot of things for me, I like visually … I like to see things in front of me, I like things being laid out clear as day.^1^ | Positive |
|  | QG | P13: I also really like a calendar part a lot.^1^ | Positive |
|  | QG | P06: I love the tracking feature. | Positive |
|  | QG | P23: I think it’s nice that you have [a] section of the app where you can … go to … the last place that you smoked because … personally, I smoke in … the same places a lot … That is kind of … cool to realize because you're like, “Oh, wait, I actually like have been staying in the same spot,” and maybe that's why … I'm always … going through all these cigarettes because I'm just in the same spot. | Positive |
|  | QG | P13: I don't like the line graph … I think it's really confusing, but I do like the calendar.^1^ | Neutral |
|  | QG | P13: The colors [for tracking are] kinda, I don't know.^1^ | Neutral |
|  | QG | P13: I like the idea of a line graph, but I didn't really understand what that line graph had anything to do with anything. I didn't like that either, a lot.^1^ | Neutral |
|  | QG | P12: I didn't like the red color background [for tracking cravings]. I thought that was a little bit … like an alarm is going off.^1^ | Negative |
|  | QG | P11: I don't quite understand [the mood icons and] what it tracks.^1^ | Negative |
|  | QG | P14: It's clearly not an easy thing to do, the fact that you’re clicking “I slipped” which is red and has a big X on it … Especially if you feel like you're doing it quite often while utilizing the app.^1^ | Negative |
|  | QJ | P14: I think … [QuitGuide] actually included a drop-down selection menu and it had points where you chose the reason [for slipping] and if I remember correctly … [I] wondered if there was an option to enter other [reasons for slipping] … [to] be able to truly dictate your reason … [in] words if it wasn't there [in the drop down menu] … [QuitJourney] incorporates that so I think that that's great. | Positive |
|  | QJ | P04: I like the look and … that you can put your own reasoning [for slipping] versus a generic … drop-down box. You actually [can] get to the root of … why you [slipped].^1^ | Positive |
|  | QJ | P12: I like the look of this [app’s “tracking slips” page] … better [than QuitGuide’s].^1^ | Positive |
|  | QJ | P25: I think [tracking] does a really good job of just keeping me accountable and also engaged with the app. I like how it tracks your mood, and it asks you the reason for slipping. I like how it [asks] … if you're happy, sad or in the middle. | Positive |
|  | QJ | P30: I also like the mood [tracking] part. Any time of the day … I could just put how I'm feeling. | Positive |
|  | QJ | P02: I was just gonna say it seems engaging … I don't really … understand the scale for my mood, but… I feel like it makes me wanna … try it every time … I have a craving.^1^ | Neutral |
|  | QJ | P17: [The mood tracker] looks pretty bland … like when … you go to the doctor and they ask you to [move the scale] … It looks … something like that.^1^ | Negative |
|  | QJ | P37: This [tracking slips page] … to me feels like it's the end of a journey, rather than like a step … It's like an end form in a way … to me.^1^ | Negative |
|  | QJ | P17: What I least like about [the app] is like the scales [to track your mood] … [they’re] kinda like that doctors’ survey … they give you. | Negative |

Participant ID appears before each quote for attribution.
QG = Quit Guide, QJ = Quit Journey.
^1^Indicates quote mentions design concepts.
^2^Indicates quote mentions intent/willingness to use.
