# Supplementary material for: A feature-based qualitative assessment of smoking cessation mobile applications
Source: PLOS Digit Health. 2024 Nov 21;3(11):e0000658. doi: 10.1371/journal.pdig.0000658 (PMC11581403; doi:10.1371/journal.pdig.0000658)
Supplement: S4 Table — (DOCX) [file pdig.0000658.s006.docx]

**S4 Table. Themes and illustrative quotes of individuals who smoke on the tailored feedback and support feature in QuitGuide and Quit Journey**

| **Themes** | **App** | **Quotations** | **Sentiment** |
| --- | --- | --- | --- |
| Performance Expectancy | QG | P24: I think [personalization] helps because you maybe form habits [and] you don't notice you're doing it, but if you realize how often you go to a particular place, like a balcony, you might say, okay, this is kind of … redundant, let me do something else with myself. | Positive |
|  | QG | P22: [Personalization] maybe makes you aware of triggers you had that you didn't even realize. | Positive |
|  | QG | P11: I think [personalization] would be really helpful … I love this idea, especially the … location and time of day, if it can track that and … send you messages. | Positive |
|  | QG | P13: I like the [personalized] time of day [notifications] the most. The location [notifications are] nice, and I like that … I like the time [notifications] because certain times make me want to kind of smoke … so [it] would be nice to be able to set that up and have reminders not to, or things that I could do during those times. | Positive |
|  | QG | P13: If it tracks time it would be really, really useful for me, especially if it's able to … actually give me … warnings during that time and loud or like in-your-face warnings. That would be really good. If I was going to quit smoking this would be very useful, just because of the time thing. | Positive |
|  | QG | P11: I think [personalization] would be … super helpful and useful, especially with the notifications and even … the locations stuff, because I think that's something that … I don't tend to realize … Going somewhere specific might be … a place where I smoke more often and that's just something that I don't realize. So, like being more mindful and aware of it would help me to quit easier. | Positive |
|  | QG | P13: Overall, I just think [personalization is] more like a useful thing … it's going to help me do something. | Positive |
|  | QG | P13: [Personalization] would be useful to use like if you're quitting. | Positive |
|  | QG | P05: I love [personalization] because it's letting you make … your plan to quit. It's not just … a broad … quick fix … Everyone's different, you know? So, it's not like, oh, do this and it'll work. It lets you actually kind of tailor it to your specific needs and your habits rather than just being a kind of … generalization. | Positive |
|  | QG | P06: It would be really useful … having the option to tailor the app … to what I like and just to personalize it. Just being able to personalize the app could help me get tailored help and recommendations … In the long run, it will be … more helpful than building a generalized app. This one [app] at least feels more personalized, and it's really awesome. | Positive |
|  | QG | P06: [Personalization] will be useful. | Positive |
|  | QG | P20: I think [personalization is] a wonderful idea … It just gets more down to the root … It handles it more … thoroughly … just down to the root, really. | Positive |
|  | QG | P21: I really like [personalization] because … my journeying to quitting smoking isn't the same as … the other participants … My triggers could be completely different than the other person … just little things like that. It's not a one-size-fits-all, and I think … [it] treats quitting smoking or any type of addiction as if it's … one way to do it and that's it … or there's one triggering [and] if you just get rid of that … it's done. | Positive |
|  | QG | P21: I know like with other … habit [trackers] … you can count your days, so … if you did use it for smoking you can probably count as many days as you didn't smoke … Having this geared toward smoking, I think that that, you know, is beneficial. | Positive |
|  | QG | P14: I agree, I think [personalization is] a great idea. Really great, cool to have a place within the app that will be utilized and is useful. | Positive |
|  | QG | P03: I think for me, it all goes back to the accountability … I know me, personally, I smoke a cigarette first thing in the morning … when I wake up regardless [of] what time that is … If it went off and was like, good morning … and then they knew that I was about to smoke a cigarette … because I had put that in the app … if you're able to do something like that … then it would make me accountable for it to where I wouldn't end up smoking that cigarette first thing in the morning … then get me to the point where I get so used to not smoking when I first wake up, that it’s not even on my mind no more. | Positive |
|  | QG | P03: Let's say … I forgot that I had put the fact that … I had the court date coming … [and] it completely … left my mind … but the app hasn’t forgotten … Here I go in my in my purse to go grab a cigarette or whatever, and here goes my alarm, “Oh, no, don't do that.” | Positive |
|  | QG | P01: [Personalization] would definitely be helpful. | Positive |
|  | QG | P10: I feel like with most apps, it's always nice to kind of personalize them because it makes you feel more connected to it. So if you [are] … trying really hard to quit, it could be nice to have the option to make the app more kind of tuned into … the exact way that you want to quit or the exact timeline that you want to quit. | Positive |
|  | QG | P17: [Personalization] can help … if you have certain triggers and you’re ready to smoke, it might be able to … give you some tips to try something different other than smoking. | Positive |
|  | QG | P03: Let's say … we was going to start trying this app out like later today … Well, my fiancé is having trial … Now, let's say I put that in my … quit guide app … It knows that … so, here's X, Y, and Z to do so you don't smoke a cigarette. Like, that's just awesome. | Positive |
|  | QG | P11: I think how … you can make it really personalized and … have all the data on there for … really specific things about when you are having cravings or triggers and … have that data there [is the thing I like best about the app], especially like I said the location stuff. I think also … the fact there are so many features, you can really try them all out and see which ones work for you … Sometimes you might think that something might not work or you try it and then it does, or you might think that something would work and then you get like bored of it, but you can like go do something else on there. I think that's cool. | Positive |
|  | QG | P04: Personally, if I had my reason for quitting and I had like a goal or a picture of something, like if you had kids if that was your reason for quitting, and you could put it there [on the main page], that might be helpful. | Positive |
|  | QG | P11: If you know what time of day you … usually start craving a cigarette. Then, you can … kind of mindfully choose a different activity to do that might … distract you | Positive |
|  | QG | P15: I mean, [personalization] might be a little helpful, but I don't know. | Neutral |
|  | QG | P08: Most of the time when I'm smoking, it's when … I'm on my long drives or driving to work or driving back from work … When I'm in the car that's … when I'm chain smoking, basically. So I don't know if [tailored feedback based on location] would be necessarily helpful for me because I would just, if I do get notifications … would get pinged every time I'm … driving to work or something. The whole ride I’m smoking, basically. So I don't know how helpful that would be for me to tell me where I'm smoking. For some people it might be helpful because … a lot of smokers, they get stressed out at work. And so, maybe that's the highest point of smoking or at home, so that might be their highest peak in smoking. But I don't think for me … that would be very helpful. | Neutral |
|  | QG | P12: I don't know how much [personalization] would help me because … if I really want a cigarette I'm just going to smoke it, and then I might just get annoyed at the phone. | Negative |
|  | QG | P14: I mean, I can sit here and say that [personalization] would be useful at times … I do know that life and work and how busy or hectic of a day I have. I will admit to you know, saying I did it … But that doesn't always happen. And it kind of feeds into a lot of what you'll hear with just some excuses and ways people make themselves feel a little bit better about struggling to quit. | Negative |
|  | QG | P09: I like the [tailored feedback based on location] feature. I mean, if I got into something like that and I can pinpoint exactly where I smoke … The thing is, I smoke more while I'm driving, not more so at home. I don't need an app to … show me where … I smoke because it’s like once [a] craving hits, it hits … and then I just pull out a cigarette … If my phone vibrates and reminds me to not smoke [a] cigarette, it may or may not help me put that …cigarette down, because I already have that cigarette in my hand and I'm already addicted to it. | Negative |
|  | QJ | P34: Receiving a notification at these places or at those times some way somehow, rather than if it's a message or something … that would be good. | Positive |
|  | QJ | P37: I do like [personalization] sometimes … It would be kind of nice to have … slots for places like work or school or … hints almost. That kind of stuff helps me a lot. | Positive |
|  | QJ | P35: I think, for me, I would want something … just like [at] the beginning of the app … that makes you kinda think of … where do you smoke the most? … Then, inputting those places, and then [putting] in the address, like … school, work … Then, every time I'm at [one of] those places, it would be nice to … get something that pops up, like, “Hey, maybe … cut down today, since you are at that place.” … Being able to edit that message, I think that would be really helpful for me. | Positive |
|  | QJ | P34: I do like the fact that [personalization] gets your mind going … It gets … you thinking about other stuff other than smoking. | Positive |
|  | QJ | P11: I think [personalization is] a really cool idea and could be really helpful … I like how … you can add as many as you want, or have as little as you want. | Positive |
|  | QJ | P31: [Personalization] would be helpful. | Positive |
|  | QJ | P04: I think [personalization would be helpful] cause no two people are the same. So, what works for one person, somebody else might need a little adjustment to it for it to work for them or be able to be a better use for them. | Positive |
|  | QJ | P14: I think [personalization] would be beneficial, and I like the customized … criteria options that each user can input into their app. | Positive |
|  | QJ | P14: [Personalization is] more beneficial and necessary and very helpful, tool wise. | Positive |
|  | QJ | P04: I think … [personalization] would be beneficial, yeah, to be able to track [my location and times]. | Positive |
|  | QJ | P08: I think the notification thing is actually pretty nice, because I won't have notifications just randomly coming in on my phone, I can actually set a time. So, that way I know … this isn't the time to get a notification, or … this is the time. | Positive |
|  | QJ | P10: Yeah, I think [personalization] is very useful, too. | Positive |
|  | QJ | P10: I think it's great because everyone's cravings are different … it could give you a better idea … if you smoke in one place a lot, if you can get a notification saying that, hey, you're in that place right now so be careful. That could help, I guess. | Positive |
|  | QJ | P02: Yeah, I like [personalization] too. I like that it like preps you … to be able to deal with, you know that, that temptation … to just overlook it and be strong. | Positive |
|  | QJ | P16: I think [personalization] would be very useful for me. | Positive |
|  | QJ | P02: I think [personalization is] a great way to like stave me off of … the temptation should I encounter … considering … it’s a chronic thing, and you know, this place that I go to. | Positive |
|  | QJ | P08: Yeah, I think [personalization] would be really, really useful. | Positive |
|  | QJ | P28: I can see it being helpful, too, especially if you smoke when you're around a certain group and a certain area. It can be something to pull you out of that routine and make you think twice about it, or especially … if you have a certain time of the day when you go and you do a break, it can maybe give you a second thought on that instead of you just going ahead and doing it. | Positive |
|  | QJ | P13: I think [personalization] would be … good. But I think it would only be … really good if you [could] set multiple locations, and maybe more times than two … I think [it would be] a cool idea … for a while, while you have it. You could maybe say, oh, I smoked at this time and this location, and over time, maybe you have … a thing that kind of shows you what times are the highest chance you're smoking, or what locations … you’re smoking [the most] … Sometimes you don't notice, and that would probably help. | Positive |
|  | QJ | P28: I think it would be really helpful if you could put as many locations and times as you want, like an unlimited thing. [If] it saves all of that, and it manages to detect when any of those is happening, and if it kept track of it. | Positive |
|  | QJ | P29: I find that I often smoke at work. So, it would be pretty useful to have an app on my phone that … knows when I'm at work, and I get, like, a little notification that's like, hey, you're here right now, you usually smoke, but today don't do that. | Positive |
|  | QJ | P01: [The mood tracking] could be really good for like a work situation where you usually smoke at a certain time … and it can record and try to remind you that you're going to be okay the next day … around this time. | Positive |
|  | QJ | P04: I think that you should be able to add more [locations], for sure … What if … going to a ballgame or … just something random … is another stressor. So, you should be able to be able to put those down … ‘Cause if you weren't able to, then you wouldn't be tracking properly, and it wouldn't actually be beneficial to you. | Neutral |
|  | QJ | P30: I think [personalization] would be pretty useful … I feel like it would have been more applicable to me back in high school. If I could have [set] something like that to a certain friend's house or something … because we all used to … one spot, usually … If I saw like a notification, like a popup, that … might have given me more incentive to not smoke. | Neutral |
|  | QJ | P17: [It is] like too many things to track … Going as far as tracking my location to let me know … when I need help to stop smoking … it may be a little too much. I don’t know, but I guess it's ok if I wanted to do that. | Negative |
|  | QJ | P25: I think the notifications [aren’t] that accurate, because you can always have cravings, different places, different times. So, it's not really a specific time, you have a craving or a specific place, like you can see someone smoking or watch TV and you … have a craving. So, the notifications … doesn't really do much. | Negative |
| Effort Expectancy | QG | P19: I get where [personalization] could be easy to use. | Positive |
|  | QG | P13: I was going to say, [personalization] sounds really easy to me. I mean, I didn't even have … a smartphone for a very long time so … I only use very simple apps because I'm kind of slow with this kind of stuff and it seems extremely easy to me. | Positive |
|  | QG | P14: Easy, [personalization] doesn’t look difficult. | Positive |
|  | QG | P03: Just the fact that you can set it to your own ways of life … and it's not just the app … going based off of … everybody in the world that smokes a cigarette … you can personalize it to where … you can get it to your life [and] schedule … [to] help you personally. | Positive |
|  | QG | P07: It's kind of hard to say, like [if] I was at work and I'm in a rush or something like that, I only have a couple minutes to come outside while I’m taking out the trash or something … [so] usually I'm probably not going to be grabbing my phone or [if it] vibrates, I'm not going to hear it … If I'm just sitting at home ... I'm on my phone a little bit more so. | Neutral |
|  | QG | P23: If it kind of helps with my lifestyle … I would have to try it first and see how it works personally for me. | Neutral |
|  | QG | P19: I feel like … if you're gonna go out in public and you're gonna be going to a store, or work, or whatever you're going to … you're probably gonna be doing that socially, like with your friends … As soon as … that thing goes off on … your reminders … and it alerts you, you're gonna be like as quick as you can turn it off … cause you’re busy. You're with your friends or you're doing whatever … [and] you don't want someone dinging on you. | Negative |
|  | QJ | P31: [Personalization] looks pretty easy. | Positive |
|  | QJ | P35: [Personalization] doesn't look challenging at all. Very simple like most of the apps that we do use regularly. | Positive |
|  | QJ | P14: Yes, I think [personalization] seems very easy. | Positive |
|  | QJ | P08: [Personalization] looks pretty easy to me, like just by looking at the layout of the app, it looks pretty self-explanatory.^1^ | Positive |
|  | QJ | P13: [Personalization is] easy to do, and only takes a few seconds. | Positive |
|  | QJ | P29: [Personalization] seems pretty straightforward. | Positive |
|  | QJ | P17: I don’t know, it’s … too much going on right now. The location and … stuff like that. | Negative |
| Facilitating Conditions | QG | P12: I wouldn't say that I dislike the idea [of personalization]. I just couldn't see myself doing it, [I] just don't feel like it. | Negative |
| Hedonic Motivation | QG | P05: I would kind of try to use it … if I was craving a cigarette … [So] the alternative would be like, oh, well, let me get on on quit guide instead … so every time I would want to smoke … [I would] try to … switch [to] the habit of … pulling out the phone and then … personalizing a little bit where you are, what you're craving or anything like that | Positive |
|  | QG | P22: I think [personalization] might be fun if … you’re a couple of days into quitting smoking and feeling successful. But I think in the times you weren't feeling successful, it wouldn't be fun. | Neutral |
|  | QG | P13: I mean, I don’t know if fun would be like the right word. | Neutral |
|  | QG | P11: It's like less of a game than other apps are, but I still think this is something like … fun in its nerdy way. | Neutral |
|  | QG | P11: I think [personalization] would be like interesting … I really like data stuff so that would be … kind of fun in a weird way. But I don't think it would be like a game. | Neutral |
|  | QG | P04: I think it could depend on … the person … using it … If I were to use [the app?] and I was in a stressful place or something … maybe it would calm my nerves … To say this is what I want to do but knowing that I have this app … it can kind of … replace that and I don’t know, make it a little more of a game to quit smoking … rather than just being like oh, I can't smoke cause I can’t smoke. | Neutral |
|  | QG | P15: I don’t know about fun. | Neutral |
|  | QG | P01: I don’t know. I really... I don't know. I'm not sure that I could call [personalization] fun? I mean I don’t know … I’m still undecided on it. | Neutral |
|  | QG | P01: Yeah, I think [personalization] has the possibility to be fun, but I mean I'm not sure. | Neutral |
|  | QG | P07: It'd be fun for somebody that is really trying to quit and would really take this app seriously. [Someone who] would even you know, maybe even set reminders and things like that to come up on [their] phone. Some people don't like that, but I feel like if you're constantly kind of reminded … through the app … then you know it'd be a little bit more. I guess I wouldn’t use the word fun. | Neutral |
|  | QG | P24: I think [personalization] will be fun, especially … if you like did it with someone else and you guys can … keep up with each [other] and … how the app is making you feel instead of just doing it by yourself. | Neutral |
|  | QG | P13: Not really like oh, [personalization] is not? fun. | Negative |
|  | QG | P06: But I don't think [personalization would] be fun. | Negative |
|  | QJ | P37: I think that [personalization] could be very helpful and fun in terms of this app just because … I honestly don't often think about what, where or when my triggers could happen. So it might be … kind of fun to map out and see if this can keep me to it. | Positive |
|  | QJ | P31: I don’t know if [personalization would] be fun. | Neutral |
|  | QJ | P14: I don't know that [personalization] would be fun, maybe interesting to see patterns. | Neutral |
|  | QJ | P04: I think it just kind of depends on peoples’ definition of fun. | Neutral |
|  | QJ | P02: I don't know about fun … I think [personalization] could be annoying but … it's necessary. | Neutral |
|  | QJ | P13: I don't see [personalization] as fun, unless it has … active things that I would consider fun. Like depending on how fun the game … [or] is there a challenge or whatever. It [would] be more of a routine thing that I would have to commit myself to if I was serious about this. | Neutral |
|  | QJ | P13: As for … fun, it just depends … on … what kind of games you'd have or challenges and things like that | Neutral |
|  | QJ | P13: But at the same time, [personalization is] not fun, because it's the thing that commits yourself to … checking it. | Negative |
| Not applicable | QG | P02: I think [personalization will] be a real cool feature just … knowing if something's coming up … and how you can … just …personalize it. I just think it’s … a real cool feature, it just connects well. | Positive |
|  | QG | P11: I love that, I think personalized stuff is always really interesting | Positive |
|  | QG | P21: So, I think that the personalizing is probably what would set … this app apart from others. | Positive |
|  | QG | P02: I like the positive reinforcement that I feel … you could get from [personalization] … I think that's … really engaging | Positive |
|  | QG | P18: That's pretty awesome, like … if [the app] can alert you or let you know … don’t smoke a cigarette or say something that gets you thinking … maybe I should [not] smoke right now or today. I think that's cool. That's the start, you know? | Positive |
|  | QG | P11: I think that'd be cool if … you did have track my location on and [there] was like a feature where … then if you're at someone's house or … outside of work or something … it like can tell and it's like, hey, I just want to like, you know, give you an extra positive message or something … The time of day [support] I think is really cool. | Positive |
|  | QG | P13: [I like the] the time of day [messages] and all that stuff. | Positive |
|  | QG | P13: Customization is really important, in my opinion, like, with anything. | Positive |
|  | QG | P05: What I like best [about the app] is just that you can kind of personalize it to yourself and your habit, because it's not just like … reading a guide on how to quit smoking or anything where it's going to tell everyone that same thing. I like how you can actually kind of get it to suit your specific needs. | Positive |
|  | QG | P01: I like the ability to personalize [the app]. I think that could really, really make it … work for everybody. | Positive |
|  | QG | P04: I like [the app] because … you kinda tailor it to your own needs or what you want out of it versus other programs where you have to stick to … a certain rightness … It's very modernized for I guess today’s day and age | Positive |
|  | QG | P11: The option to do more if you want to is good to have. Like, if you want to write about [your reason for quitting] or like some people love little journal diary things … So, having that be there if you want to, but yeah not being necessary. | Positive |
|  | QG | P14: Um, so I do like the fact that … after you click this trigger option, it brings you to that next screen which has those four little bubbles that give you additional resources to utilize because you've slipped, which is you know requesting help.^1^ | Positive |
|  | QG | P01: I like the idea of [personalization]. I just don't know … if I would follow through with it [and] how long I would actually stick with it. | Neutral |
|  | QJ | P36: I think [personalization is] awesome. | Positive |
|  | QJ | P34: [Personalization is] a good feature. | Positive |
|  | QJ | P12: [Personalization] seems like a good option to have. | Positive |
|  | QJ | P11: I like the different options [of what you can do after tracking a slip]. I think it's cool because the person can pick what they want to do. It's kind of frustrating when you can only do like one thing. | Positive |
|  | QJ | P13: I really like the location [personalized support] a lot. | Positive |
|  | QJ | P25: I actually like how there's a set date for you to quit. It's not just today. It gives you a timeframe to work on it. | Positive |
|  | QJ | P30: I think [being able to ask the app for help at a location or time] is a pretty good feature. | Positive |

Participant ID appears before each quote for attribution.
QG = Quit Guide, QJ = Quit Journey.
^1^Indicates quote mentions design concepts.
