# Supplementary material for: A feature-based qualitative assessment of smoking cessation mobile applications
Source: PLOS Digit Health. 2024 Nov 21;3(11):e0000658. doi: 10.1371/journal.pdig.0000658 (PMC11581403; doi:10.1371/journal.pdig.0000658)
Supplement: S5 Table — (DOCX) [file pdig.0000658.s007.docx]

**S5 Table. Themes and illustrative quotes of individuals who smoke on the educational content feature in QuitGuide and Quit Journey**

| **Themes** | **App** | **Quotations** | **Sentiment** |
| --- | --- | --- | --- |
| Performance Expectancy | QG | P22: I really like that. I feel like, when you're quitting, you need as much support and information because everything around you is telling you to smoke. | Positive |
|  | QG | P24: [The cessation information] will help you like, know, get distracted, I guess. | Positive |
|  | QG | P22: [The cessation information] provides you a way to … get more into your quitting. | Positive |
|  | QG | P23: I think that's nice also. I feel like a lot of resources is good … That's pretty important. | Positive |
|  | QG | P24: I feel like it's good to have … information on the side … sometimes [you] can't convince yourself that it's not healthy. It can show you … the rates of how many people get to certain illnesses and maybe … teach you to be more worried about yourself compared to just the fact that nicotine is addictive. But it can show you what the actual cigarettes are doing to your body. | Positive |
|  | QG | P12: [The cessation information] is good … Sometimes that's what you need to see in that moment when you're needing some help. | Positive |
|  | QG | P11: I think information is always really helpful. | Positive |
|  | QJ | P38: I like [the cessation information]. I think it gives you a little more information, and … it doesn't make you feel bad … if you slip up … It gives you a little more information and makes you feel comfortable about the journey. | Positive |
|  | QJ | P31: The information I think is very helpful. It makes you feel like you're not alone once you go through some of this stuff. | Positive |
|  | QJ | P11: I think it'd be really useful to have all this … information … of like one thing. | Positive |
|  | QJ | P17: I think [cessation information] is very helpful. | Positive |
|  | QJ | P02: I think [cessation information] is just a great add on, because there's so many things … we're always looking at. It's just something else … just to help us out along the way | Positive |
|  | QJ | P16: I think it’s useful because the app is primarily just for smoking. Whereas if you go like on Google or something you can search [for information] and they give you all these other resources … This is like your own personal journey to … to quit smoking. So, I think it will be helpful. | Positive |
|  | QJ | P08: It would be super … resourceful and educational. | Positive |
|  | QJ | P16: I think that [cessation information] will be useful. | Positive |
|  | QJ | P26: I think it's good to have [information] … on stand-by … I like how it’s … just like a resource, you can just use at any given time, rather than if you don't have the patience. | Positive |
|  | QJ | P26: I think it will be very useful because it's more direct, rather than somebody just looking up on the Internet ways to quit. I feel like this app can give you a lot more direct information rather than just [what is] scattered on the web. | Positive |
|  | QJ | P30: I kinda like how [the information is] organized, how there's like a structure to it. There's five steps, but I feel like those five steps won't always work the way it tells you to.^1^ | Neutral |
|  | QJ | P29: I think that this is all useful information, but it looks like a lot of information that I already know. | Neutral |
|  | QJ | P28: I honestly think this [information] would be good for … a beginner person that doesn't really know all these things, because then they can learn. But most of this … is stuff I've already read and seen millions of times … The idea of going back to this one section, every time you need help, is kinda slim because … all you're doing is reading the same thing over and over | Neutral |
|  | QJ | P33: I think [the information is] kind of vague and … it doesn't seem like it offers that much … I'm sure when I might go … within the app, that would do more than just being like, “Hey, don’t quit.” … You should do that too, but … if it's gonna have like a database [or] a tutorial, it should have more than that [and be] more specific. | Negative |
|  | QJ | P33: Personally, I don't like [the information] just because it's not very specific. I don't think it would do much for me. | Negative |
|  | QJ | P28: What I dislike is that [this information] might be a lot to read for some people … The way that it set up, they might not, you know, do things that way. So, for them, it might not be [helpful]. It might not be helpful for everybody, but the intent is good, and I like that. | Negative |
|  | QJ | P13: I can see how [this information] could be useful to some people … but I don’t really think it's … useful to me because I will probably just look elsewhere information … [I] probably wouldn’t … go to this section of the app, to be honest. | Negative |
| Effort Expectancy | QG | P24: [Viewing cessation information] looks super easy. | Positive |
|  | QG | P23: [Viewing cessation information] look[s] pretty easy to use. | Positive |
|  | QJ | P37: I like how centralized all this information is, I don't have to look for anything.^1^ | Positive |
|  | QJ | P14: I agree, I really like the way [the information is] laid out visually, the color codes, and I really like the fact that it allows you to drop down … I really like the way that looks and it seems very easy to jump around in.^1^ | Positive |
|  | QJ | P14: I definitely think it seems very easy. Like I said earlier, I like the way it allows you to go from broad topic and … then breaks down and weeds out … the elements and options based on the broad topic. So it's not necessarily hard, as far as the reference goes, and utilizing it [as] a reference guide.^1^ | Positive |
|  | QJ | P12: Yeah, [using cessation information ] looks easy. | Positive |
|  | QJ | P10: [The cessation information] seems pretty straightforward and easy to comprehend, find and use | Positive |
|  | QJ | P28: I like that the depth and the information [that] is in the app and you don't have to open a separate window [using] your Internet. That part is nice and I like that it's organized in steps.^1^ | Positive |
|  | QJ | P28: It's a simplified version of information. | Positive |
|  | QJ | P28: Definitely easy to use [cessation information]. | Positive |
| Hedonic Motivation | QG | P24: [Viewing cessation information is] not necessarily fun. | Negative |
|  | QJ | P04: I like that you go through the different numbers and … it's cool that [the five quitting steps] goes from red to blue and it kinda makes your mind believe that you’re … going down as well which is good in this case … So a little bit more fun with the colors and stuff and just information.^1^ | Positive |
|  | QJ | P31: If you’re serious about trying to stop smoking … it’s serious and fun, I mean you could make it fun. | Neutral |
|  | QJ | P08: I don't know about fun. | Neutral |
|  | QJ | P31: I wouldn’t call [cessation information] fun. | Negative |
|  | QJ | P35: Yeah, [cessation information is] not fun. | Negative |
|  | QJ | P16: I wouldn't use the word fun. | Negative |
|  | QJ | P30: No, I wouldn’t say [cessation information] would be fun. | Negative |
|  | QJ | P29: It's just like [cessation] information, so it's not really what I would call fun. | Negative |
|  | QJ | P33: I wouldn't call [cessation information] fun. | Negative |
| Not applicable | QJ | P33: [The how to quit page] looks nice. | Positive |
|  | QJ | P36: I also like that [all of the information] is all in one area.^1^ | Positive |
|  | QJ | P12: I like how [the five additional information buttons] start on red and end on blue, kinda goes down … [from] hot colors into … cool colors like you [are] progressing away from something bad.^1^ | Positive |
|  | QJ | P13: I really like how it looks. First off, I like just the colors and everything. It's very organized and clean, and I think it's good to have like tips to help you quit smoking. However, I also think that it's a lot of information for someone just to read like just to read … texts of information get kind of boring.^1^ | Neutral |

Participant ID appears before each quote for attribution.
QG = Quit Guide, QJ = Quit Journey.
^1^Indicates quote mentions design concepts.
