# Supplementary material for: A feature-based qualitative assessment of smoking cessation mobile applications
Source: PLOS Digit Health. 2024 Nov 21;3(11):e0000658. doi: 10.1371/journal.pdig.0000658 (PMC11581403; doi:10.1371/journal.pdig.0000658)
Supplement: S6 Table — (DOCX) [file pdig.0000658.s008.docx]

**S6 Table. Themes and illustrative quotes of overall perceptions of QuitGuide and Quit Journey, names, and landing pages**

| **Main Theme** | **Subtheme** | **App** | **Quotations** | **Sentiment** |
| --- | --- | --- | --- | --- |
| App Name | Not applicable | QG | P16: [QuitGuide] was a cool name. | Positive |
|  |  | QG | P17: [The app name is] cool. | Positive |
|  |  | QG | P10: [The name makes me think of a] guide to quit smoking quickly. | Positive |
|  |  | QG | P22: I like [the name] … I like that. It tells you there’s guidance for you. So … I feel better about having guidance. | Positive |
|  |  | QG | P06: It's [a] pretty simple name and it’s straightforward. I think it's very easy to understand … They can get … from the name that it’s about quitting and … a guide for quitting … I think it’s good. | Positive |
|  |  | QG | P05: I like [the name]. It's … simple and straightforward, and it says the purpose of the app in the name ... If you were to recommend it to a friend or a colleague who is also trying to quit it's easy for them to remember so they can … go search for it later … It's good that it states the purpose of it in the title as well. | Positive |
|  |  | QG | P20: I think … that the name is usually the first thing that you notice about an app, you know, so everyone likes guides. | Positive |
|  |  | QG | P21: I think it sounds like a good name. Especially with apps, I think almost like the shorter, more catchy the name, the better and I think that … sums up what you're trying to, what the whole app is about, but in like a short, little … way. | Positive |
|  |  | QG | P07: Comes to mind that … there's … goals to accomplish … Maybe just the name kind of pops out, like maybe it's a little bit user-friendly or not user-friendly … As a guide … if you follow steps it could work, but it's like one those things where you probably have to be pretty committed to it full on, 100%. | Neutral |
|  |  | QG | P04: I like the name a lot, but it kinda makes me think that it has, like, other options besides smoking, like if I wanted to quit drinking or something that … it would be a guide for … whatever I wanted to quit. | Neutral |
|  |  | QG | P02: I think [the name] is cool … It's OK. It's not like over the top … It's sensible | Neutral |
|  |  | QG | P18: I'm not sure [about the name], because like is it an app or a handbook or? … What is it, like is it something that you read or something that you scroll through … that's the hard thing I'm trying to figure out. | Negative |
|  |  | QG | P19: I wouldn't know what the app was going to be about without reading the description. I definitely wouldn't think that it would be about smoking … The name just kinda throws me off. | Negative |
|  |  | QG | P24: [The name is] very vague, it could be like a quit guide for people who have … like all types of addiction. | Negative |
|  |  | QG | P23: [The name] doesn't sound too specific to smoking. | Negative |
|  |  | QG | P13: [The name is] not very memorable and … it sounds like a lot of other things too … Like unless I was … actually using the app, like a lot, like I'd probably forget the name of it. | Negative |
|  |  | QG | P11: [The name] sounds like a lot of other things, so I think … it doesn't stand out as much … Quit Guide I think, doesn't really explain that it's smoking specific because you can quit lots of things. | Negative |
|  |  | QG | P12: [The name is] not super memorable. | Negative |
|  |  | QG | P14: I kind of hear that name and I think something that’s so broad, that if I were to come across it I would assume that it was something strictly to utilize for reference. I'm thinking like work-related, policy, and procedure document and guides on how to do this or how to do that … I feel like I would be more prone to notice it or come across it and think it would be applicable to me in my life for trying to quit smoking if it incorporated and had something that was more descriptive of what … the purpose is. | Negative |
|  |  | QG | P16: No, I don't really care for [the name]. | Negative |
|  |  | QG | P02: I don't know [about the name]. Not much to it. | Negative |
|  |  | QG | P01: [The name is] not terribly catchy. | Negative |
|  |  | QG | P13: I don't like the name. | Negative |
|  |  | QG | P03: When I hear the name Quit Guide I think, like, a quick fix …You know, I think if I was to tell [people that wanted to use the app], hey, there's an app called quick fix, they probably wouldn't want to use it because they think, oh, well, it's a quick fix, that's not going to work for me. | Negative |
|  |  | QG | P08: If I'm looking for an app, I want some something that I know [is] directed towards smokers … or … something kind of specific to the situation that I'm in. | Negative |
|  |  | QJ | P38: Honestly, I think it sounds a little more inspiring, to get you … to want to quit. You know … Quit Journey … it sounds a little better, a bit more exciting. | Positive |
|  |  | QJ | P17: I was interested [in the app]. Like you want to try [to go] on a journey to do something good for you | Positive |
|  |  | QJ | P25: I actually like the name. I think it's very catchy and I believe that quitting is a journey. It's a long journey so it's eye catching to me. It makes me want to try it [to] see what it's about and if it's helpful.^2^ | Positive |
|  |  | QJ | P34: If they make it appealing, I guess [Quit Journey is] a good name. | Neutral |
|  |  | QJ | P37: I think that it's kind of … a good image with the journey word in there. But I think as the name for an app, [it] could … fall short. I think … it would need to be explained pretty immediately after hearing it to get the context of it. | Neutral |
|  |  | QJ | P14: I like [the name]. I think that the word journey kind of indicates that like most of us who've tried quitting or have quit, we understand that it's not a quick process and … the name … entails it's a journey and … it's a long one sometimes for most of us. I think it's kinda catchy. But I guess for me, if I came across it … it wouldn't indicate that it was catered to smoking in any way. So I might … not be quick to [get it] unless it yielded a result within my actually seeking out … [a] quit smoking app in my search [bar]. I wouldn't necessarily click on it if it was in a big, random … chunk of apps for me to look into. I wouldn't think that it had anything to do with smoking, but I do like it. | Neutral |
|  |  | QJ | P36: I personally think [the name Quit Journey is] a little cheesy. | Negative |
|  |  | QJ | P31: I don’t particularly like the name [Quit Journey]. It sounds like someone else's story. | Negative |
|  |  | QJ | P35: [The name] feels a bit corny as well. I like the idea, I do. I mean the other ones, I saw … [were] quit now [and] I mean, you can't just quit that easily. So … I like the word journey because it is a journey from the beginning to the end. But I just don't like that name right away, I think they should have something catchier. | Negative |
|  |  | QJ | P12: [The name Quit Journey] doesn't really jump out to me. I think it's … not enough, [it] doesn't do much for me. | Negative |
|  |  | QJ | P08: I think the name doesn't like specify … what it's for … People may think it's for different things. … the name doesn't sound specifically [for] … smoking to me. | Negative |
|  |  | QJ | P13: I personally don't like it too much to be honest. To me it seems like it would be hard to remember unless you use it all the time. I do like that it has quit in it … I don’t know, quit journey is … I don't know [if I] really like that too much. | Negative |
|  |  | QJ | P28: I also like that it says quit, but the whole word journey sounds like it's going to be a tedious process, like it's going to be a frustrating one that takes way too long. | Negative |
|  |  | QJ | P29: I like that quit is in the name, but I think that with Quit Journey, it's not very clear that it's about smoking specifically. So if I was to see it in the app store, I wouldn't necessarily be drawn to it because I wouldn't really be sure … what it is about. | Negative |
| App Landing Page | Performance Expectancy | QJ | P26: And it's just a tally up of like what you did today, so then you could just immediately … click on the button that you did, depending on what you [are] feeling or what the situation was. | Positive |
|  |  | QJ | P13: [I] also liked the bottom parts [of the screen], where I'm guessing like you can do something like play a game or something when you're craving [and] get your mind off of that. I like that, as well. I think it would be really helpful.^1^ | Positive |
|  |  | QJ | P28: I like that, it seems like there are distractions … I’m one of those people who are really hard on themselves. So, I would probably just focus on the fact that I slipped all day long, and if there's distractions, that's really helpful. | Positive |
|  | Effort Expectancy | QG | P07: I mean [the landing page] kinda looks like it's user friendly and kinda is personalized to you.^1^ | Positive |
|  |  | QG | P04: I think [the landing page] looks very … professional and … it looks easy to use just on this … first page. It doesn’t make you feel … completely bummed if you slipped up. You can just say I slipped or I was smoke free today. I like that.^1^ | Positive |
|  |  | QG | P05: I think [the landing page is] really straightforward. It looks good to me. It looks like it would be really easy for just about anyone, whether or not you're really tech savvy to pick up [and] use.^1^ | Positive |
|  |  | QG | P09: I mean [the landing page is] pretty simple.^1^ | Positive |
|  |  | QG | P14: I visually like it … sometimes I think that simplicity is better, especially for … a landing home screen … because it is simple, it's not too crazy. I feel like apps should be set up personally and what I like is [for them] to be laid out in ways where you can dive further by clicking … another field or clicking down to populate other options that are embedded within it, so I do visually like the way it looks.^1^ | Positive |
|  |  | QG | P05: Yeah, I like [the landing page] … It's streamlined and simple. I can already tell what all of the options to click on there do.^1^ | Positive |
|  |  | QG | P21: Honestly, I didn't think I was gonna … like the main screen as much as I did but I think that it's simple.^1^ | Positive |
|  |  | QG | P12: I like the simplicity of [the landing page] … [just] two options … to pick between there and [it’s] just simple.^1^ | Positive |
|  |  | QJ | P26: I think it's great. I feel like if it's me personally … if there's more too much, it’s … just gonna [get] thrown away. Throws off the whole point of just the app itself … I feel like it’s supposed to be just self-explanatory. | Positive |
|  |  | QJ | P08: I was actually in one of the previous sessions and you guys had … displayed a different version [of the app landing page] and I actually like this version much more. I think it's much more … easier than the last one.^1^ | Positive |
|  |  | QJ | P30: I think [the landing page] looks pretty good … It's pretty simple [and] I think … that simplicity is the … the good part.^1^ | Positive |
|  |  | QJ | P28: I like the design. I like that it's easy to understand and read everything. Nothing is… hard to look at, the colors are bright, but I agree [with] the craving button. It kind of feels like it's mocking me.^1^ | Neutral |
|  | Facilitating Conditions | QG | P09: This is almost the same kind of app that I had when I was trying to quit drinking. You know it gives you your mood, I mean it didn’t track the craving by any means. | Neutral |
|  |  | QJ | P30: [I] actually think that [the landing page has] a good kind of interface … it's really similar to the Fitbit app … with all the little kind of buttons on the bottom to go over to each page … I like how it looks.^1^ | Positive |
|  | Not applicable | QG | P02: I think [the landing page is] colorful. It looks … legit … [and] professional … not like this kid type of app. Very … clinical is the word I'm trying to use.^1^ | Positive |
|  |  | QG | P01: I like the colors [of the landing page] and I like the layout.^1^ | Positive |
|  |  | QG | P23: [The landing page] looks pretty clean.^1^ | Positive |
|  |  | QG | P24: Looks like the colors are not too elementary [on the landing page], it doesn't seem … like a playful app, like it seems serious.^1^ | Positive |
|  |  | QG | P11: I really like it all. | Positive |
|  |  | QG | P21: I like [the landing page]. I really like the … top part where it says … I was smoke-free today, or I slipped.^1^ | Positive |
|  |  | QG | P06: This [landing page], it's amazing, it's awesome. | Positive |
|  |  | QG | P05: I like [the landing page]. It's really streamlined.^1^ | Positive |
|  |  | QG | P21: I think that [it] would be really cool if I could like, put a picture of my daughter there, so it says my reason for quitting and had a picture of my daughter. I think that would be really cool. Make it more personable. | Positive |
|  |  | QG | P02: I was just looking at the layout, it’s just colorful, but not too colorful. I love the blackish gray background … it seems like it's something that I could just immediately click on … to break my habit, probably.^1^ | Positive |
|  |  | QG | P20: This makes me think that even if you slip up, like it's okay, like let's keep going … let's deal with the mess up today and just … keep stepping. | Positive |
|  |  | QG | P13: I like the idea of … “I was smokefree today” and “I slipped” kinda thing … [but] I would also feel like demoralized a bit. I like that idea. I'm not too big on the green color and stuff.^1^ | Neutral |
|  |  | QG | P21: Maybe the layout looks a little boring, I guess like [with] the dark colors … I don't really know what that picture is supposed to be where it says “My reason for quitting.”^1^ | Negative |
|  |  | QG | P05: The text underneath the “My reasons for quitting” [on the landing page] looks a tad bit small. I can't quite read what it says.^1^ | Negative |
|  |  | QG | P13: In my opinion, [the landing page] kind of seems kinda depressing a bit … because I'm not like that big into these types of [dark] colors … I’m more, you know, a brighter person.^1^ | Negative |
|  |  | QG | P13: [The landing page] doesn't seem very bright and doesn't seem … very welcoming as well … Like, those types of colors.^1^ | Negative |
|  |  | QG | P12: I think [the landing page] looks just kind of not very well coordinated with the colors. Like it's just visually … doesn't look like anything in particular … I mean, they got the blue for smoke free and the red for not, so that's kinda like a good-bad type thing. But then they have orange and green down there at the bottom, and I just feel like we've got like four colors here and it doesn't need to be that many.^1^ | Negative |
|  |  | QG | P12: [The landing page] just looked kind of clunky and … it just didn't look very well done to me.^1^ | Negative |
|  |  | QG | P21: So [the landing page] does just look kind of dark [and] … bland … That’s a lot of a reason … if it doesn't catch your eye and it … doesn't hold your attention, you're not going to use it as long nine times out of ten.^1^ | Negative |
|  |  | QG | P09: I mean there's not really much to [the landing page]. | Negative |
|  |  | QG | P21: I guess the only thing I don't really like is the X [icon] because … if you're trying to quit at all, it's a good thing … It almost seems … like a fail … I guess in a way that if you did smoke and you're trying to quit, it is a fail, but … I feel like just the fact that … you're trying to quit is like a positive [thing] in itself.^1^ | Negative |
|  |  | QG | P12: I don't like the colors [of the landing page].^1^ | Negative |
|  |  | QJ | P11: I like [the landing page] a lot. I really like the color scheme besides the red for [the] “I slipped today” [button]. But … the mountains are cute and how it shows the sky being … clear … that just … makes me think of how if I quit smoking, I'll be able to … do more outside things because my lungs won’t be so shitty.^1^ | Positive |
|  |  | QJ | P34: [I] like the colors and everything, how it's schemed together.^1^ | Positive |
|  |  | QJ | P12: I like this [landing page] better than [QuitGuide’s] for sure.^1^ | Positive |
|  |  | QJ | P14: I do like the way … [the landing page] looks.^1^ | Positive |
|  |  | QJ | P17: I like the layout of the [landing page’s] screen.^1^ | Positive |
|  |  | QJ | P04: I like the “I’m craving” button. I don't know what it does, but I like it.^1^ | Positive |
|  |  | QJ | P10: I think [the landing page] looks pretty stylish. I like the colors a lot.^1^ | Positive |
|  |  | QJ | P26: I like … all the options [on the landing page] because … when you do smoke a cigarette, those are things that you do think about, at least … [for] me.^1^ | Positive |
|  |  | QJ | P13: I like that [the landing page is] … bright and I like [that] the background … you know … [with] hills and everything is more bright. I also like the options. I don't really like that craving button too much, but I also like the red “I slipped today” [button]… and the blue “I was smoke free today” [button]. I think it looks really nice color wise.^1^ | Positive |
|  |  | QJ | P35: For me, I definitely like the layout [of the landing page] a lot and I do love the colors, but … the “I slipped today” [button] in red, … for some reason … it doesn't make me feel comfortable.^1^ | Neutral |
|  |  | QJ | P12: I like the buttons on this [landing page] and I like the image at the top with the mountains because that's like the journey … But they kinda look like they don't really go together.^1^ | Neutral |
|  |  | QJ | P02: I just think [the landing page is] not really bold and I don't like the color. I don't … think I really like the color scheme.^1^ | Negative |
|  |  | QJ | P13: I feel like [the craving button is] just off from … everything else… Because everything else looks … like it fits and then you just have like that [button] and it doesn’t really fit.^1^ | Negative |
|  |  | QJ | P38: To me, it makes me feel like once you press the red [“I slipped today”] button, you might … get in trouble or something. It seems kind of … bad.^1^ | Negative |
| App in General | Performance Expectancy | QG | P14: [The app] might be helpful for those individuals that are 50 and 60 that didn't grow up in the era that the younger people are. | Positive |
|  |  | QG | P16: [The app] looks very helpful. | Positive |
|  |  | QG | P18: I think [the app] would be helpful and useful. It’s something new, something that people probably haven't seen before … I think it can be very helpful, because, like I said, for me, I really want to stop smoking. | Positive |
|  |  | QG | P18: I definitely like to try new things out because you never know, they could be helpful … and [the app] could give you some good pointers and stuff.^2^ | Positive |
|  |  | QG | P19: [I would want to try the app to quit or] slow down, because if you can see … how much … you're consuming then you can … think like, whoa, ok, slow down a little bit.^2^ | Positive |
|  |  | QG | P08: Yeah, [the app] would be pretty beneficial … I believe, because I like live on my phone and work on my phone a lot, so it could be beneficial to me. | Positive |
|  |  | QG | P08: I would probably use this as well, because … I've never tried an app for quitting smoking, but it seems that would be more beneficial to me than like a nicotine patch or like all of the other stuff. So, I would definitely use it.^2^ | Positive |
|  |  | QG | P22: I like with the app … more ways in which it's showing me my progress and keeping me encouraged and positive … as much as possible … It's like if you screw up, just like positive stuff and not starting you all over, I think that's a good thing in the app. … It's like, oh, you were at 30 days, and now you're back at zero because that can be very discouraging. Like, keeping it encouraging when you screw up and then also encouraging you throughout and then showing where you're at. Like, whether it's like health wise, or maybe mentally, this is how it's helped you so far, just scientifically. | Positive |
|  |  | QG | P22: I do like the ways how you can … personalize [the app] … if you're that into it. Like, if you wanna be tracking where you are and how you're feeling, and that kind of stuff. | Positive |
|  |  | QG | P22: I think it's good that [the app] gives you a lot of information … Instead of … like using the patch … [where] you're not that connected with the quitting. It makes you feel a little more, maybe, like, in control, or something, just like putting all the responsibility on the patch, you know. | Positive |
|  |  | QG | P24: I just think [the app is] interesting … Some people have been smoking for like five years, I haven't reached that bar. So, I think it's kinda cool that around my … era there's a way that my phone could probably help me quit smoking instead of me just leaving it up to my surroundings, my company, my willpower. It kind of helps me feel like … I'll have that progress and that data to show that I quit before, so I would never need to start again and go through all that again. So, I think … I would appreciate that. | Positive |
|  |  | QG | P23: I think I would give [the app] a try, also, just because … I've had like kind of a hard time … trying to quit smoking … about a year now. I've been smoking for quite a few years, but I've just been trying to … fully quit recently and it's been like pretty hard … so I think trying out this app could help out a little bit.^2^ | Positive |
|  |  | QG | P11: I think [the app is] super cool. I would love to try it out … I have never looked for an app that's to help people stop smoking cigarettes specifically … I do think it's like a lot more hands-on and interactive and like personalized, which is really cool … I would be excited to try it and I think it'd be helpful.^2^ | Positive |
|  |  | QG | P13: The main thing I really like [about the app] is … it keeps track of like you're craving times and could possibly like send you warnings or quotes or something like that. I really like that. | Positive |
|  |  | QG | P06: [The app] could be useful in the long run. | Positive |
|  |  | QG | P04: I would definitely use the app … I think it kinda gives you different options to … kinda deter your mind from wanting to smoke but also to kinda hold you accountable for yourself and … you get to work on your own time. You get to set your own goals and stuff like that that, so I really like that, I would definitely use it as well.^2^ | Positive |
|  |  | QG | P04: [The app is a] good incentive for me and it would hold me accountable I think … because it would be … like my program, I guess.^2^ | Positive |
|  |  | QG | P21: I think that [the app] has like, it's unique, and I think that, you know, I just I think that there's a lot of really good ideas with it, and I think it can be really helpful to a lot of people. | Positive |
|  |  | QG | P16: Just from being on the phone with you guys … and just all the layouts I think it's [a] really great app. Like, I would definitely get the app … I'm already excited about it right now because, like I said, I've been trying to stop smoking cigarettes. So, I'm … excited already.^2^ | Positive |
|  |  | QG | P16: I would … use [the app] because … I think it's really great and … just from the little demonstration you've given us so far it seems like it will be helpful. It may work for some; it may not work for others. But for the ones that have worked for, I think that it is useful to us, and I will definitely … download the app and use it.^2^ | Positive |
|  |  | QG | P14: It seems like [the app] has a lot of features that are effective and useful for [quitting] … [like] additional reminder and just positivity that can really be necessary for some people that need that, that struggle to really try to quit, like myself. So, I definitely would use it. I can think of a few people at the top of my head that would love it as well, so I think it’s great.^2^ | Positive |
|  |  | QG | P02: I think that [the app] just looks professional … like a legit app … It seems like … It’s something that I could truly rely on to track what's going on … and when I have those cravings … to get some empowerment to stop it somehow.^1^ | Positive |
|  |  | QG | P05: I would definitely use [the app] because I've tried to quit before … to no avail … This, honestly, almost makes me excited to try quitting again … which I have wanted to do. This is definitely more and more incentive for accountability, and it's just a different approach, so it's trying something new.^2^ | Positive |
|  |  | QJ | P34: I feel like [the app is] definitely different than anything, any quitting app that they have out now. Will … definitely be a game changer, for sure. | Positive |
|  |  | QJ | P37: I definitely would try [the app], especially … [for] having my own kind of handle on my health, especially now and in the near future, I think would make me feel really good.^2^ | Positive |
|  |  | QJ | P26: I think [the app is] very well-thought-out and engaging … I think it’s very… modern. So, I think … [if] this app was to be created it can give a lot of help … especially to the younger generation … rather than just not thinking that there's no hope at all … I feel like the app will just give them more of a sense of … okay, like, you know, I can do this. | Positive |
|  |  | QJ | P02: I'm a very technical and analytical person and I really, really like … the different things that [the app is] telling me, like how much time saved, spent, avoided … Everything like that … kind of droves out my psyche that … I'm making a difference. | Positive |
|  |  | QJ | P10: I'm pretty excited. I think it looks like a great app, and [has] a lot of good features, and it seems better than anything that I've ever seen for quitting smoking. So, I think it's … gonna be really good. | Positive |
|  |  | QJ | P28: This one [app] definitely wins in the unique department, and it seems like they came up with more than just saying, hey, don't do this. There's things to keep … you … engaged and actually [have] a way to track how you're doing. | Positive |
|  |  | QJ | P13: I'd use [the app] mainly because I feel like it would actually help me keep track of things and help with cravings and have locations [to] notify me and stuff like that, and that might really help me keep on track and remember … [that] this is what I wanted to do and kind of motivate me a lot more.^2^ | Positive |
|  |  | QJ | P08: I think at least for me [I would engage with the app] … for all the toxins to be out of my body and … for me not to have those cravings anymore … [The app] can last up to like 3, 4 months … After that I know that the app might be forgotten | Neutral |
|  |  | QJ | P01: I think with a couple of tweaks [the app] … could be very helpful. | Neutral |
|  | Effort Expectancy | QG | P16: I think [the app] will be easy [to use] … I feel like it would be easy. Because … the sample , it looks easy. You know just from looking at it, it doesn't seem like it would be hard to figure out how to use the app … so it looks really easy for me. | Positive |
|  |  | QG | P14: I completely agree [that the app looks easy] … Sometimes simplicity and keeping it minimal with what is absolutely necessary instead of trying to add all these additional features and maneuvering mechanisms … I think sometimes that can also hinder people from wanting to really utilize it and take advantage, because it's just so busy and full of so many options. And viewing it on a mobile phone with all of that going on can be extremely overwhelming. | Positive |
|  |  | QG | P14: The simplicity and the ease of access feel that I'm getting from a lot of this. | Positive |
|  |  | QG | P10: [The app] seems pretty straightforward to me and pretty easy to understand. So, I feel like … you could get used to it pretty quick. | Positive |
|  |  | QG | P22: I like [the app] … It looked clean and easy to use just from the main page … When you open an app … for the first time, if it looks too complicated when you open it, you're just like, oh, just delete it. So that, it looks simple to use.^1^ | Positive |
|  |  | QG | P06: [The app] seems really simple, it's really easy to use. | Positive |
|  |  | QG | P05: I like [the app], I think that it definitely looks streamlined and easy to pick up … It's something that I personally would definitely use.^2^ | Positive |
|  |  | QG | P04: [The app] seems really easy and I think once … everything is worked out with … the kinks or whatever, then I think it would be super easy to use. | Positive |
|  |  | QG | P05: [The app is] very simple and straightforward. | Positive |
|  |  | QG | P14: I would use the app, I think. Like I said earlier, I think it is simple.^2^ | Positive |
|  |  | QG | P17: [The app] might be easy to use. | Neutral |
|  |  | QJ | P14: I think [the app is] way better visually and seems much more user-friendly the way it's laid out … compared to what I recall the last focus group included … It makes so much more sense and … like I said, just way better, visually appealing, and easier to click through.^1^ | Positive |
|  |  | QJ | P17: I like that [the app] is easy to access. | Positive |
|  |  | QJ | P16: I think [the app is] easy [to understand and use]. | Positive |
|  |  | QJ | P08: [The app] looks pretty easy to me. | Positive |
|  |  | QJ | P10: I think [the app is] very easy looking. | Positive |
|  |  | QJ | P02: [The app is] totally easy [to understand and use]. | Positive |
|  |  | QJ | P13: I think [the app is] a great idea, and I think a lot of it's super easy to use, simple, easy to understand as well … Maybe some small things added would help, but, overall, I like it a lot. | Positive |
|  |  | QJ | P13: The thing I like most about the app is just the overall organization of it and how … easy it looks to use.^1^ | Positive |
|  |  | QJ | P30: I like … the user interface the most and … the simplicity of it.^1^ | Positive |
|  |  | QJ | P28: I really like the interface [of the app]. I like that it's simple, it's easy to understand and easy to use, and when you click on things it doesn't take a whole lot of time.^1^ | Positive |
|  |  | QJ | P13: [The app seems] extremely easy. | Positive |
|  |  | QJ | P28: [The app is] pretty straightforward. | Positive |
|  |  | QJ | P30: I think [the app is] really simple to use, and I’d probably use it.^2^ | Positive |
|  | Facilitating Conditions | QG | P09: I'd use [the app] because I want to quit smoking … Something I've always wanted to do is quit, because my health is gonna go downhill if I keep smoking … So, maybe this will help me, maybe.^2^ | Positive |
|  |  | QG | *(In response to question about app’s compatibility with one’s life):* P24: I think so. I use apps a lot. I feel like I'm always downloading one any other day. | Positive |
|  |  | QG | P22: The thought of cancer is so scary … so it’s like I would really want the app. | Positive |
|  |  | QG | P19: I feel like for younger people that smoke, it would be a better thing for them than it would for older people that smoke because older people that smoke are gonna be set in their ways … they're not putting down their cigarettes … But [for] younger people, that might be an app for them. | Neutral |
|  |  | QG | P16: I mean … all of us in this day and age, we’re all phone savvy and … I'm really good with phones … For the older crowd that's not really phone savvy … that would be my only concern. But for me, I am phone savvy. | Neutral |
|  |  | QG | P23: I think [engagement with the app] all depends on … how serious someone is about quitting smoking. | Neutral |
|  |  | QG | P21: I think based on everything … we talked about today; I think that I'm definitely … looking forward to the app’s release … I guess price is kind of a big thing, but … I definitely would get the app if it was … in my budget, I can definitely say that I would.^2^ | Neutral |
|  |  | QG | P12: For me, personally, I don't think I would really use [the app] because I want to spend less time on my phone, not more … I just kinda get annoyed when I feel like my phone is telling me to do stuff, like I just want to live my own life and use the phone when I need it … Overall, I kinda like don't always trust my phone all the time because I feel like I use it too much.^2^ | Negative |
|  |  | QJ | P08: I've tried a couple of smoking apps, and I think this is definitely the best one I've heard of. | Positive |
|  |  | QJ | P29: I think [the app] is [better than other smoking cessation programs] … I texted this phone number that was like a quitting smoking hotline, and I just kinda did it cause I was bored. I wasn't super interested in quitting at the time but … it didn't feel personal at all … It was just a bot … giving me like generic messages … I feel like this app is very unique in the way that it is so personal. So, I think it definitely has a leg up on other quitting smoking apps. | Positive |
|  |  | QJ | P16: I think something like this [should be] free. We [are] already spending all of our money on cigarettes anyway. So, I don't think that people should be … because … that makes people … numbers … So, I think it being free, or at least like a trial or something at first, but I'm all for free. | Neutral |
|  | Hedonic Motivation | QG | P09: It’d be fun to actually have that app get me to quit 100%, that would be fun. | Positive |
|  |  | QJ | P33: It's hard for me to call a quitting app fun. | Negative |
|  | Social Influence | QG | P07: If I saw that [the app] was working for [another person], I might be interested [in using it too]. | Neutral |
|  |  | QG | P09: If [the app] was working for [another person], it’d be beneficial for me, and I’d try it.^2^ | Neutral |
|  |  | QG | P08: I would have to see if [the app] worked with [another person]. Then, I would definitely use it.^2^ | Neutral |
|  |  | QG | P10: If I saw someone using [the app] and that it was doing well for them, then I would want to try.^2^ | Neutral |
|  |  | QG | P04: If my friends are super interested in like an app or game or something like that and I see it working for them then … that's gonna kind of pique my interest as well … Like If I had it … I would be like, hey, my friend, look at this and they were like, oh, yeah, let me try it. So, word of mouth and just, like, influence and stuff. | Neutral |
|  |  | QJ | P16: If [the app is] working for [another person], or if I was hearing … good things about it, yeah [I would use the app too].^2^ | Neutral |
|  |  | QJ | P13: I think that if [another person] brought [the app] up to me, I would definitely think about it. Maybe if they wanted me to use it, I’d think about it and download it, and might use it … I'd be more likely to.^2^ | Neutral |
|  |  | QJ | P28: I would definitely give [the app] a try [if another person was using it]. I'll try … things like that at least once.^2^ | Neutral |
|  | Not applicable | QG | P19: I think [the app is] a good idea. It would be a good idea for a lot of people … Overall, I think it's actually … a good thought. | Positive |
|  |  | QG | P17: Yeah, I think that [the app is] better [than other smoking cessation programs]. | Positive |
|  |  | QG | P08: Yeah, I would try [the app].^2^ | Positive |
|  |  | QG | P09: Definitely give [the app] a whack, for sure.^2^ | Positive |
|  |  | QG | P23: I feel like [the app] looked pretty clean and like sleek. It’s got like a nice look to it.^1^ | Positive |
|  |  | QG | P23: I think I'd try [the app].^2^ | Positive |
|  |  | QG | P24: I would definitely try [the app].^2^ | Positive |
|  |  | QG | P05: I will definitely give [the app] a shot.^2^ | Positive |
|  |  | QG | P05: Honestly, [the app is] really interesting … I was curious when I just heard that it was a smoking cessation app, but knowing a little bit more about it and seeing it now, it's just really neat. I like pretty much everything about it. | Positive |
|  |  | QG | P03: I like [the app]. I'm interested in using it and seeing what it's about and seeing how good it would be to help me to stop smoking, see if I can get my mom and stepdad to use it to try to stop too.^2^ | Positive |
|  |  | QG | P02: I was just saying I feel like [the app] connects you … on a real level, and I'm not just saying that. | Positive |
|  |  | QG | P03: I think it's a pretty good app and I’m kinda intrigued to use it and see what it's about for sure.^2^ | Positive |
|  |  | QG | P04: I'm excited for [the app] to come [out]. | Positive |
|  |  | QG | P13: I think that extra features would be like really cool as well. Like, especially [tracking your smoking] locations, or I like the idea that you're able to write [your] triggers down, like, oh I was doing this and this is why I smoked. That's really cool. | Positive |
|  |  | QG | P13: I don't like the colors too much either.^1^ | Negative |
|  |  | QG | P15: I don't think I would use [the app].^2^ | Negative |
|  |  | QJ | P35: I really like the idea. I haven't really seen … other apps. I've just seen … the layout and stuff … Besides making some tweaks to it … I do like what I saw … it seems interactive with what I would want and not too pushy or anything. I think it just needs like … color and just like tweaks here and there, but I would definitely use [the app].^1,2^ | Positive |
|  |  | QJ | P31: I think I'd be inclined to try [the app].^2^ | Positive |
|  |  | QJ | P14: I love [the app]. I think it's great. | Positive |
|  |  | QJ | P12: [The new app is] definitely an improvement over the [previous version] we looked at. | Positive |
|  |  | QJ | P12: [The app] looks better, just kinda makes more sense than the last [version] did. | Positive |
|  |  | QJ | P14: I think my favorite piece [of the app] is the homepage and the ways that y’all incorporated everything and changed the layout of that [from the previous version].^1^ | Positive |
|  |  | QJ | P16: I've been waiting for the app to come out.^2^ | Positive |
|  |  | QJ | P10: I feel like [the app] has a lot of good feature sets. | Positive |
|  |  | QJ | P08: I like the idea of the app. I think … when the app comes out, [I] would love to download it and … see how it works for me … I think you guys have a lot of ideas that other apps towards smoking, or even other habits, I don't think that they've ever implemented it, so I think it might be a really good idea.^2^ | Positive |
|  |  | QJ | P26: I like the layout [of the app].^1^ | Positive |
|  |  | QJ | P08: I liked … the general layout of the app.^1^ | Positive |
|  |  | QJ | P08: I know that I would definitely download [the app] when it came out.^2^ | Positive |
|  |  | QJ | P16: I'm willing to download [the app].^2^ | Positive |
|  |  | QJ | P25: I would love to download [the app]. Also, I think that every day that I do smoke a cigarette I always think about ways to quit smoking. So, I would love to try it.^2^ | Positive |
|  |  | QJ | P02: That's like the only [app] that I've really seen that that seems engaging, seems like I would actually use it.^2^ | Positive |
|  |  | QJ | *(In response to question about app’s compatibility with one’s life):* P26: I would say so. Just like the display, like the options that we can pick as to what we did in a day, like the money saved, the time [spent] smoke free. Like, those are things that I think about on the daily [and] when I’m about to smoke a cigarette for sure. | Positive |
|  |  | QJ | P26: I think [the app] can definitely change the way that … I overlook [factors] on trying to quit smoking. | Positive |
|  |  | QJ | P08: Overall, I think it's … just a great app idea. I don't think anyone has ever implemented something like this to help people stop smoking. So, I think it's a really great idea. | Positive |
|  |  | QJ | P16: I think that it's a good app, because, just for someone to care about people that have an addiction, because smoking is an addiction … Like, it makes it a good app, because … you're around a lot of people that smoke, and that never will stop smoking. So, just to have people create an app, to help people fight the addiction, which a lot of people don't have that support, I think it's really great. | Positive |
|  |  | QJ | P28: I really like [the app] … aside from the name and the one or two things we said that we'd like to change. I think it's a great idea, and it has good intentions, and it could be great. | Positive |
|  |  | QJ | P30: I think there's a lot of potential too [with the app]. | Positive |
|  |  | QJ | P29: What I like the most [about the app] is definitely the interface.^1^ | Positive |
|  |  | QJ | P13: I do like how [the app] looks, it looks a lot better [than the previous version] in my opinion.^1^ | Positive |
|  |  | QJ | P13: I think [the app] looks more welcoming, more inviting, more bright. I think things are more clear and more organized [compared to the previous version]. I just think it looks better overall. It's 100% improvement.^1^ | Positive |
|  |  | QJ | P01: I like the layout, the layout’s designed a lot better than the last [app].^1^ | Positive |
|  |  | QJ | P28: I would try [the app] too.^2^ | Positive |
|  |  | QJ | P27: I think [the app is] very well designed and [I’m] actually more impressed than I thought it was going to be.^1^ | Positive |
|  |  | QJ | P34: I think the concept [of the app] is good … I feel like there’s a little bit of spacing [issues] going on and … the colors and all that [could be modified]. Good stuff, but I like the energy behind it.^1^ | Neutral |
|  |  | QJ | P31: I think the idea [of the app] is good and I think as you go along you get the kinks out of it … [and] if something that needs to be added or taken out I think that’ll happen, but I think it's a good concept and it has a lot of informative information. | Neutral |
|  |  | QJ | P04: From the last [version]… it seems like they've worked out … their kinks and made things a little bit better, which is awesome … I'm sure there's still a few things they have to figure out, but … I like the overall view of [the app]. | Neutral |
|  |  | QJ | P08: If I had one thing to change … [it] would be … the general layout … I don't know if it's very … appealing to the eye.^1^ | Negative |
|  |  | QJ | P27: The layout is just a little bit too relaxed and the way things are nowadays things that are really bold and flashier are what seem to work better.^1^ | Negative |

Participant ID appears before each quote for attribution.
Total number of quotes for app name = 39. There was a total of 25 quotes for QuitGuide app name (negative = 14, neutral = 3, positive = 8). There was a total of 14 quotes for Quit Journey app name (negative = 8, neutral = 3, positive = 3).

Total number of quotes for landing page = 53. There was a total of 31 quotes for QuitGuide app landing page (negative = 10, neutral = 2, positive = 19). There was a total of 22 quotes for Quit Journey landing page (negative = 3, neutral = 3, positive = 16).

Total number of quotes for app in general = 125. There was a total of 64 general quotes on QuitGuide (negative = 3, neutral = 10, positive = 51). There was a total of 61 general quotes on Quit Journey (negative = 3, neutral = 9, positive = 49).

^1^Indicates quote mentions design concepts.
^2^Indicates quote mentions intent/willingness to use.
