# Supplementary material for: A feature-based qualitative assessment of smoking cessation mobile applications
Source: PLOS Digit Health. 2024 Nov 21;3(11):e0000658. doi: 10.1371/journal.pdig.0000658 (PMC11581403; doi:10.1371/journal.pdig.0000658)
Supplement: S7 Table — (DOCX) [file pdig.0000658.s009.docx]

**S7 Table.** **Illustrative quotes of suggestions to improve QuitGuide and Quit Journey**

| **Theme** | **App** | **Quotations** |
| --- | --- | --- |
| Self-monitoring | QG | P05: Do you know if it has … statistics? So, you can go back and see, because I think it would be most useful if you could notice trends, like if on a certain day you were a little higher stress and then your cravings were higher that day or vice versa. It’d be helpful to be able to see like patterns as to what stimulates your cravings more and what maybe makes them not as bad. I think that'd be really beneficial to try to quit, because then you would know maybe certain situations or places to avoid and things like that. |
|  | QG | P03: If I was able to track my cravings and I had like an alarm … go off … then that would help me … Like, oh, you're about to get a craving, so I know you’re about to smoke a cigarette … so, here's an alarm [or] hey, do this instead … Then, it would get my mind off of wanting to smoke that cigarette. 6. |
|  | QG | P22: I'd like if the app … gave you encouragement … as to why it was helpful … Like, we think this is helpful because of this and then it kind of pushes you … Oh, OK, well, now I want to track that as this app said, oh, people that track it have this much more success rate … Some incentive to do the extra work of tracking. |
|  | QG | P11: Maybe … after you click the … “I [slipped]” [button] … there could even be like a quick popup screen that's like, you know, we know this isn't easy, and then just like reminding people that like slipping is a normal part of recovery, but … you can still get back on track and … don't beat yourself up about it and then it would … minimize … and then go to the screen where then you would … fill out all your information.^*^ |
|  | QG | P12: They could integrate [the tracking data] with … your calendar maybe. I would be more likely to look at it. |
|  | QG | P13: I think I would like [the visualization options] better if it was just like, here's the data on when you messed up kind of thing. |
|  | QG | P11: Maybe [adding] something where it's like … something [triggering] you to slip … [or] slipping is a normal part of quitting … Like, you know, it's not the end of the world and it's common.^*^ |
|  | QG | P13: Motivational kind of things would be really nice [to include with messages after a slip].^*^ |
|  | QG | P11: One thing though, is … if you have to write about the trigger … Something that stopped me from using other apps is if you … have to fill out that box before you can submit it and … sometimes I just want to say … I had a lapse or something and don't want to … write about it. So … having the … text that you write about it be an optional thing, and then even if I'm … busy or something, I can go back and fill it out later. |
|  | QG | P13: Another thing, it'd be nice if you could click on the days [of the calendar] and see … maybe just the times that you slipped like maybe you didn't slip all day, but just like a time period, or maybe you could write in the calendar … if you clicked a date or something. |
|  | QG | P11: I think the calendar stuff is cool … [but] it's like, if it was just like one slip versus like the whole day … like one cigarette versus … if you start smoking a whole pack for … a day that's … totally different … Maybe having that there, so you don't feel like it was … a total failure if you have … one puff of a cigarette … compared to … a whole pack. |
|  | QG | P11: If [the calendar] had … the past month and it was like … you had like a number of cigarettes … If you did lapse it could be like zero and then you have like three in January but like … two in March or whatever. |
|  | QG | P14: Are these [visualization features] laid out in ways in which you can specify by entering your date range? … If it's like a journey that I'm taking … or if I'm continuously struggling over a 2-, 3-month period, [I would want] to see more than just a month layout or have it analyzed … on the line graph where you can see … was it conditional to that week or … the holidays or working overtime at work for those two weeks … [I would want] more than a 30-day timeframe … I'm not quite sure if this app is kind of built in a way that you're catering to users that you hope won’t need that long of a time frame of utilizing the app … like, say, six months. |
|  | QG | P14: [There should be] like built-in options [in the visualizations] for you to like drop-down and click January 2019, or even allow you to click and check boxes, and click January, February, or say it's today … I want to see how I've done since the holidays and I wanted to see my progress and what had been going on since January 1, 2020 and until now. |
|  | QG | P23: Advice or … words of encouragement … [I] would like [after tracking a slip]. It's something that would be … important.^*^ |
|  | QG | P22: I was hoping … if I pressed the “I slipped” button …there [would be] … words of encouragement because once you grow up smoking … you kinda just want to run away and then start again in like three months … When you screw up, I would … like … some encouragement or something like that.^*^ |
|  | QJ | P11: [It would be good idea to include more mood options because] sometimes … you might not just be … good or bad but like angry versus … sad or something and that's an important thing to … distinguish. |
|  | QJ | P32: I wish that … [the tracking slips page] had more colors, it just has too much white. |
|  | QJ | P36: [The tracking slips page] looks like a results landing page a little bit, … [so] maybe if it … [had] like a mountain theme in the back … [of] the title or something |
|  | QJ | P31: I just hope they have … the angry mood or something in … [the mood scale on the tracking slips page so you can] go from one to the other [and] just incorporate a little bit more |
|  | QJ | P34: If … [the tracking slips page] is the screen that's supposed to [be] … the try again screen … [you should] put a mountain in the background [to] make the journey continue … Just not making this screen just like the end game. |
|  | QJ | P11: It should not require you to like type much for the reason [for slipping]. I know some things … [where] you have to type a certain amount of words or whatever. But you should be able … [to] leave it blank if you just don't feel like it. |
|  | QJ | P36: It would be kinda cool if the screen was also populated with a list of feelings that aren't just happy or sad … somewhere in the middle. [It] might be interesting to see if … you're angry [or] you’re lonely. |
|  | QJ | P37: [You could add] “really stressed out”, that's not on the [mood] scale. |
|  | QJ | P11: Especially coming from the home screen, where it's like colorful and … [is] pretty … maybe … incorporate some of that … [into the tracking slips page]. |
|  | QJ | P35: For me, [the tracking slips page] is missing … like a pop of color because right now it's a little boring, so I think I want something maybe … more interactive. |
|  | QJ | P11: [Continuing the mountain background on the slip tracking page to] make the journey continue, because that also ties in with the app name if that is something they keep. |
|  | QJ | P38: I think [the tracking slips page] needs a little more color, a little more animation |
| Tailored feedback and support | QG | P22: [Having] like a personalized questionnaire, like why do you want to quit smoking? … What don't you like about smoking? Something … you could look back on that was in your words and you’re forgetting when you're really craving a cigarette or something. … When you're really craving, and then you're reading other people's words on how to quit, I don't know … When it's in your own words and you can refer back to it, that’d be something that would be useful for me … Oh, this was, in my own words, this was something I wanted. |
|  | QG | P09: I would hope [the location-based support] would be synced around maps [application]. |
|  | QG | P11: Definitely, I think that [time-specific notifications are] really important … Having it be a notification on your phone, maybe even … there's … a setting you could change where you could have it be like a more intense notification. Like the app would open … at a certain time of day or … you couldn't just like shush the notification or like move it away, but keep it like if you wanted to make it … more intense you could or be more like in your face. |
|  | QG | P11: I think pins on the map could be cool or like little icons … showing where. Maybe, if you can … rate your craving at a location it could have different … colors … on the map, to kind of … correlate with that. |
|  | QG | P14: I think that [creating your own affirmations is a] great idea and I feel like a lot of people can think about things that are funny that are applicable to their lives. Like think of what your Aunt Linda would say, or you know, someone that they know, and you can throw in … any type of quote or comment. I think that’s a really great idea.^*^ |
|  | QG | P16: [You should be able to] create your own affirmations … where … I can write my own thing and “Hey, I'm going to do this. I can do this. I know I can quit smoking.” … where I can create my own positive note to myself … because nobody knows you like you … Sometimes a lot of people look at things and they're like, oh, you know, you know it's done by a computer, but when you customize it yourself it’s things that you want to see.^*^ |
|  | QG | P11: I think it would be interesting. If you do have the location stuff or like time of day … then later you could look at that information and see like, oh, you usually have cravings at … 4 PM, 1 PM, or like when you're here … If it can show … little dots of … where … you tend to like relapse or … smoke a cigarette then it could be … interesting to see for people who want to. It doesn't have to be like right there, but … maybe a side tab that you can go to if you want more information. |
|  | QJ | P36: Just [add] … a basic background color [to the time/location notifications page]. |
|  | QJ | P37: [Classifying the time/location tailored feedback as] notifications is a little vague, maybe … you could … [give it a name] … more personalized to this app [so the user doesn’t think it’s different notifications on] … the whole phone. |
|  | QJ | P11: I think it would be cool to just have [the time notifications] … suggest … “hey, do you need help?” [at the times you need] … Or … give you some kind of … motivational quote. |
|  | QJ | P31: I'm kind of wondering … [if there could be] some message … that you could hear something [or] you can look at [when you want a time/location notification]. |
|  | QJ | P11: I think it would also be cool if [the app could] connect to something like a Fitbit or whatever. If …you log lapses at … certain locations … it can track your location or time. Maybe … it can have a little area where it's like “do you need suggestions?” It’s gonna be like … “we've noticed that … you tend to … lapse at this time or … this location … [so] … do you need extra help then?” |
|  | QJ | P35: I like this idea [of tracking locations and times] a lot. I think … just … giving [the time/location notification page] a nice pop of color, because it's a little dull right now. But I do like the idea of knowing where you smoke the most, so you're aware of that. |
|  | QJ | P11: Just [adding] colors [to the time/location notification page] or … tying it into the homepage |
|  | QJ | P34: Maybe not [title the time/location notifications page] “notifications” … Maybe … [call it] “hotspots”. |
|  | QJ | P34: Even a message you type yourself … [could be cool to receive in time/location notifications].^*^ |
|  | QJ | P36: Like a Fitbit auto generating … places would be helpful. I think if this was somehow tied to recent locations from … your Google Maps or … Apple Maps, [it] would be really helpful. Because … I think it's really easy to forget times and places that … you're at especially if you're … in a public park, you know … public places that don't have … addresses … [So] integrate location somehow. |
|  | QJ | P04: Or even like show, “Hey, you're [at this location], don't be so stressed out, maybe play a game, or something.” I don't know. |
|  | QJ | P28: The icon's aren't like a make or … break for the app, because you can always ignore [that] … They can make it so other people can do this with you … and maybe fix it so that you can have more than one location and more than two things ... as far as time … to be notified.^*^ |
|  | QJ | P30: I feel like I should add … a little information thing maybe on … the time notification [or] location notification, just like a tiny information bubble that’s explaining what it is. Or just have a section on the app that explains whatever section you want to know about. Like what this means, what that means … just in case. |
|  | QJ | P13: I think it would be cool if there was a way to … write down why you want to quit smoking … [The app] could … give you those throughout [quitting], like a month or something to keep reminding you.^*^ |
| Educational content | QG | P11: It would be cool if … you could have … links to even more information. Something that … I get frustrated a lot on is how … it's kind of like the same little bits [of information] but nothing like really helps … Like setting a quit … Well like what helps to set a quit date? … When is a good one to have? … What do most people do? And stuff like that. Like, you know you should have one, but that's not really helpful. |
|  | QG | P13: I feel like information is good, but sometimes there's not enough information and sometimes it's not necessarily tailored to you. So, other links to other things to read would be really good, maybe even like forums where other people talk about quitting smoking or something like that, because sometimes it's really good to … see what other people are doing … or maybe you could have that on the app … That would be nice as well. |
|  | QJ | P11: If [the how to quit page] … links to other parts of the app or something and maybe if it also has [more links] if you need more information because like some of those pages did look a little blank. Then it … [can] link you somewhere or … you can … keep clicking or … interacting with it to get more if you want more. |
|  | QJ | P35: Right now, [the how to quit page is] kind of dull, so I think [adding] something more fun, like [a] better font … because I feel like [the information is] great, but … most of us [already] know what they're saying. So … something just [more fun] and a little different. Maybe offering … facts … something that makes you feel like you're not alone [and] there's other people like you trying to quit as well … [It could be] a bit more … upbeat. |
|  | QJ | P37: [The how to quit page] could use some background color … to fill up that space at the bottom of the page … some illustration or something cute. |
|  | QJ | P36: You could even do … the theme of mountains in the background [of the how to quit page] … kind of creating almost like an animation that you’re like climbing this mountain as you get to like number five or something and that. |
|  | QJ | P32: More pictures [on the how to quit page] instead of just a wall of text. |
|  | QJ | P31: I'd like to see a little bit more pictures [on the how to quit page]. |
|  | QJ | P11: I don’t know if this is included somewhere else in the app but … I liked seeing before when I was like trying to quit … “in this many weeks your levels will have gotten like this much better” … Little like statistics or something … Or … [for] each number [on the how to quit page] … if you're going to [continue to] do the landscape and … the journey thing, you could have … a different picture of … nature or something. |
|  | QJ | P34: So maybe [adding] little videos … little clips, instead of so many words [for the additional information on quitting]. |
|  | QJ | P11: Especially if … on [the additional information on quitting page] … you can click on it and interact even more and like it ties into like the other helpful functions of the app. I think it could be really cool. |
|  | QJ | P31: [Additional information on quitting would] be better with some videos and stuff, that it would be fun. |
|  | QJ | P11: I really like the idea of … an animation and like climbing up the mountain as you go through the [five additional information buttons] ... I think that's really clever. |
|  | QJ | P13: I think it would be good to also have … links … to like forums … (quit smoking forums) or … sub reddits (quit smoking sub reddits) or something like that as well. |
|  | QJ | P33: Maybe [it could be fun] if [the additional information on quitting] had something funny … like … funny images of stuff. |
|  | QJ | P29: It would be useful to have … some more personalized info [serving as the additional information on quitting]. |
|  | QJ | P27: The only thing I didn't see [on the app] was anything as far as like videos or somebody presenting … ways to quit smoking, like short videos. |
| App Name | QG | P07: I think if [the application is] for smoking … it should mention smoking somehow in the name … [and that] it is just for smoking |
|  | QG | P22: Smoking Quit Guide, something along [those lines], putting like smoking in [the name], so it's a little more nicotine [related]. |
|  | QG | P23: Like Quit Smoking Now or Get Rid of the Addiction or something like that [could be better names]. |
|  | QG | P24: Maybe [a better name would be] Guide to Quit Today. |
|  | QG | P11: I'm really bad at names but like something that has smoking in the name or like quitting cigarettes … [to] just make it more specific. |
|  | QG | P11: Honestly, Smoke-Free could be a cool name too. |
|  | QG | P13: I have no idea [what a better name would be] … but probably something to … confirm that it's smoking in the name. Like, to quit smoking or to at least … cut down on the habit or something … that is recognizable. |
|  | QG | P06: I would have … smoking before quit [in the name]. So … one could know it's about quitting smoking. It’s vague at the moment. |
|  | QJ | P31: [I would] like [the app name] to be a little bit more personal. |
|  | QJ | P35: I don’t have any ideas but just something cooler, maybe like quit X … Just … [a] catchy … short name. |
|  | QJ | P37: What about … “Quit Bit” or “Quit Kit” [for app name suggestions]. |
|  | QJ | P31: I [would] like to it to say, “My journey” … or “My Quit Journey”. [It would be] more personal. |
|  | QJ | P28: I think it should be something simple with smoking in the name. Honestly, just quit smoking might work. It has to be something I can associate with that right off the bat, otherwise I'll forget about it. |
|  | QJ | P13: I think maybe it should have something to do with … smoking in the title to know that that's what it has something to do [with]. Maybe something that's easy to remember as well … not so tedious … I can't really think of anything [because] I'm not very creative, but … I do like the quit part. |
| App Landing Page | QG | P07: [I] just feel like there should be a little bit more on [the landing page] … It'd be nice to … see some of that [tracking] data displayed on the very front screen as you come in. |
|  | QG | P13: I think it would be really cool to have like not only … the reason [for quitting] but … if I was like quitting I would look … [for] health stuff because like I kinda get freaked out about that stuff a bit and that's always in the back of my head, and that might motivate me. So, maybe being able to put like pictures or links or something as well [on that page], would be kinda cool. |
|  | QG | P13: I think … visuals helped me a lot. So, I think that [data visualizations on the landing page] would be really cool |
|  | QG | P12: Where it says “I slipped” [on the landing page], I see that they're clearly … trying to [not] be too negative, but I think they could say something even more neutral like, … on the one side I was smokefree today and [on] the other one that could … be like I wasn't to there today. |
|  | QG | P13: I think [the landing page] could be a lot more bright and happy too. |
|  | QG | P11: I think [the landing page] could be just like cleaner overall … maybe like more of … a white background … I don't know why there is like the two rings [around the “Smokefree” and “I slipped” buttons], but I think that kind of makes it look like childish … It's like … a game. |
|  | QG | P13: I think it would be cool, like how you guys have the buttons where … “I've passed this day” and then “I slipped” [on the landing page], if you had another button where like I'm really stressed or I'm like really, really craving, something that you could push … and then that could maybe give you stuff to like calm those cravings or stress as well would be pretty nice. |
|  | QG | P16: I was thinking maybe brighter colors [on the landing page], the colors are like a little dark and some people, you know, they smoke because of depression. So, … when I think of dark colors, I think, you know, depressing … So, maybe like more brighter colors or, you know, just more of a … feel-good background. |
|  | QG | P12: I think that the design of the home screen needs to be more like uniform, or more like uniform color scheme, and more uniform, more of like a streamline better design. |
|  | QG | P13: [Instead of the landing page saying “I slipped” it could say] “getting back on track” or something. |
|  | QG | P11: I think the “I slipped thing” is good [but] maybe having it not be like a negative thing so people don't feel like bad about [it]. Like, oh, I had a cigarette like, you know, so they don't give up and stuff like that.^*^ |
|  | QG | P11: I think … having the actual reason [for quitting]. Like, if you have … a value chart or something like that but having that right there [on the landing page] would be really cool. |
|  | QG | P02: I like the reason for quitting, I think it needs to be bigger, so it reminds me … the date that I put … on there so I can … look back and say … “at this time [and] at this date, this is how far I’ve come.” |
|  | QG | P06: You could maybe add some inspirational texts under the “My reasons for quitting” [on the landing page] and maybe some more images just to motivate the smoker. |
|  | QG | P14: If they could compile [my data] into … a monthly report or some type of addition to the landing page. If you wanted to see … historical data like the last 30-day overview, and you see that your trigger has been … being with other smokers and trying to … remind yourself and seeing that on the landing page then avoiding [that] trigger. |
|  | QJ | P34: I would just change the font up … at the top [of the landing page]. |
|  | QJ | P36: I feel … like the buttons at the bottom [of the landing page] don't really blend in with the color scheme of the rest of the page. Maybe like a blue sky would bring it together. |
|  | QJ | P37: I think the red [for the “I slipped today” button] is a little harsh … I think maybe … if you made like a green … “I was smoke-free” [button] and … [used] … blue for the “I slipped today” [button]. I think the red, I don't know … it's just a … little much. |
|  | QJ | P13: Maybe … a symbol that's negative [could replace the lifesaver] so you wouldn’t want to want to crave it as much. |
|  | QJ | P30: I found it a bit weird that there's an alarm clock [icon] for the “I slipped today” [button]. I feel like it could be something else. |
|  | QJ | P13: Maybe … a different picture or symbol or something [for the “craving” icon]. |
|  | QJ | P28: [You could] fit [the craving button] to the bottom of the [landing page’s] wheel [of items being tracked] … more cohesively |
|  | QJ | P28: I don't understand the point of the alarm clock [as the icon for “I slipped today”]. Now that I think about it, it should be something like … one of those stop signs with the cross through it … that could work for the craving thing too because I don't really understand the whole lifesaver [being the icon for tracking a craving] either. |
|  | QJ | P02: Up [on the] top [of the landing page], where it says … quitting and stuff … I would put like my reason [for quitting] up there or … a picture … I want to do … better respiratory health or something like that … something to remind me. |
|  | QJ | P02: [The landing page] could be a little bolder |
|  | QJ | P11: [The lifesaver as the icon for the] “I'm craving” [button] is a little weird [so] maybe you could find a better picture just because it took [a while to] see what that was. |
| App in General | QG | P11: There is also … things with … health stuff like … there's … certain data they have where … if you quit smoking for a week then … your lungs are more clear [within] like 24 hours [and] your oxygen levels turned … normal. If that kind of stuff would like pop up with how many days you've been smoke free, that could be kind of like inspiring and cool. |
|  | QG | P20: I think that it would be wonderful to offer all different types of coping skills because … a lot of people say that meditation helps them … I have ADD, so … I can't really focus and meditate, you know, as to where someone else like it solves their problems … For me … really anything, but my phone is a big distraction for me, and I know that's not really the best coping skill in the world, distraction, but in some ways, it is. |
|  | QG | P21: It might actually be kind of cool … as like a distraction thing … having like a couple little games on it … Just like simple ones, maybe like a word search … to use as like a distraction tool might be kind of cool … Like a Words with Friends style [game] … just like something like simple and easy like … there's … a ton of … Candy Crush style games … I think maybe having like three games would be kind of cool, because … think about … going on a break and all the smokers at work … are going outside … Instead of just sitting there … at least having something like that to … be hands-on might be kind of cool. |
|  | QG | P14: Maybe making [the font] bigger [within the app]… I think is also a great idea. |
|  | QG | P16: If I'm looking on my screen … and I'm older lady with visual problems … that may be difficult to kind of see, so will there be like an option to make the font bigger or smaller … for just like the older crowd, because I do have a mom, and she can barely see, and she also goes to her phases where she wants to quit smoking and I will definitely refer [her] to this app once it’s … all put together. |
|  | QG | P05: I think … [the app has] a few kinks to work out and definitely something to make it more interactive with others using the app would be what I would probably … look for the most … in terms of what to add. |
|  | QG | P05: If it had … [a] message board … I like that a lot. Like, if you have a friend that's trying to quit and you see that it's working for them, or … you just see that they have this app that their using, to monitor that, and it helps … Also, knowing that you could maybe, somehow, communicate with them through that app, have like a little community of people that are trying to quit. You know if it’s a group effort. It’s definitely gonna be easier to do than just trying to do it on your own. But I like the idea of a message board I think that would be awesome. |
|  | QG | P07: A community of … people that you can connect with in the app and maybe … talk to in a sense … like kinda get together like we are now and kind of discussing these things. |
|  | QG | P08: It's actually a really great idea if there is a way that you guys can have like a chat among like other smokers so you can get like their actual … daily life ideas of smoking and things like that. |
|  | QG | P22: It would be nice to even have like an area … [for] like asking questions. It's like almost a questionnaire for you that you can refer back to that would be … positive about why you want to quit smoking. That sort of thing. |
|  | QG | P22: Like, maybe you guys [can] have a section that had like reviews that were posted [every] so often of people that are feeling like they're liking the app, and it's helping them … Like, people using the app and they're talking about the app, not just like random people who are quitting or have quit or something. |
|  | QG | P22: I've been smoking so long, and … it's just been so daunting for me … So, just like an app, that's ultra encouraging. I know … some people do better with harsher, but for me, that can be daunting … Maybe there's a way you can edit things throughout the app. Like show me this part because this is helpful for me … Like being able to maybe personalize to that extent would be cool too or … I like this part of app, so I want to see more of this. |
|  | QG | P11: Some of [the features] I didn't understand what the point was, maybe even if there was like some button that you can click so it like explains what it's showing if it's not clear … if it's not clear then I feel like it's not really that like important for me. |
|  | QG | P06: Which I dislike about [the app] is … the absence of maybe like an online board where friends or people in your circle can post on the board. Just a simple board, not like a social media platform. Just a simple platform where friends or people in your circle can post motivations, inspirations. |
|  | QG | P13: Maybe there could be … [personal] achievements on it that could kind of make it fun for some people.^*^ |
|  | QG | P05: I agree [with] kind of … making it a game. I could see that potentially making it fun. |
|  | QJ | P33: A reward system … would be … the right idea … Something … consistent towards, like, oh, you did this … good job … You can have it … accumulate.^*^ |
|  | QJ | P36: I'm curious … if sensors are incorporated into this app, would there be like notification prompts from the app that … [are] like “hey, your heart rate is up, how are you feeling kind of thing?” Because … that might be kind of cool and … would get me to engage [with] the app because I have a lot of apps that sit on my phone unused. |
|  | QJ | P35: Facts … like “your lungs are doing so much better” or just … things like … “you’re helping with the environment” … something … interactive like that I think that would be fun for me because … I think we all like small … memes or … just facts … Those paragraphs might be a little too long for the average person. |
|  | QJ | P37: The theme of the “journey” … could honestly go … to all of these [app] pages if you guys could find a way. |
|  | QJ | P36: I really like the idea of … having … the statistics on …. your health improvements. For me, that's a really big motivator. |
|  | QJ | P17: Probably adding … rewards you know when you press on something and you actually do it. Something that might reward or might keep them interested.^*^ |
|  | QJ | P14: I think that … adding an element of incentives, in addition to … showing the user real-time tracking of how much money they've saved since starting the process … [essentially] implementing reward type of elements.^*^ |
|  | QJ | P30: I think you should add … a little … information bubble or something … So, it's like you could just tap on it and then it gives that little text box or something [to] explain what [the requesting help buttons] are. |
|  | QJ | P28: Maybe there should be … an information bubble, just so you have an idea of what the “help me” means. |
|  | QJ | P29: If it's possible, adding the feature to add friends or communicate with other people. I think that's a really important feature that, if it could be added, would be really helpful. |
|  | QJ | P29: Something I've kind of been thinking this whole time with this app, like a community aspect would be really nice |
|  | QJ | P25: I also think that your health is the most important thing. So, you guys should kind of push that issue, as far as that's the main reason why people should try to quit because of the health, first and foremost, not just the rewards and stuff. |
|  | QJ | P27: I think that's like the missing ingredient, like that social aspect where you’re connecting with others, that [can] enhance it even more and make it even better. |
|  | QJ | P16: I think [the app will] be great too. Like, if people can upload videos, maybe of their journey, they [can] choose to share it with everyone … and it’ll go to like the main feed of the app and you can … share your testimony of why you relapsed that day or why is it hard for you … because … sometimes people want to know that they're not alone in this struggle. |
|  | QJ | P25: It can be like a social app where people … who smoke … try to come together and try to quit together. Just like people smoke socially, like social smoking. So, I think it may become … a social app. More people can try to quit together, make it easier. |
|  | QJ | P25: I think you guys should add a health section where you can like track your breathing as it gets better, [like] your blood pressure, just health-wise so you can know how much you're improving by the day. |
|  | QJ | P02: I think [the app is] just a little too cute for me right now. I just want it to be a little bold and have … a bolder interface. |
|  | QJ | P16: I have a lot of Latino friends and some of them … they don't really read in English. So, I … hang around a lot of diverse people. So, [having multiple languages as] an option because … a lot of things like on your phone … you can change the language. |
|  | QJ | P36: I think it'd be kinda cool … [for the developers] to look into some mindfulness apps because … there's one called … Aura that I have looked into that … just has like a really sleek design interface and kind of integrates … all of the things that people were suggesting around … the way that you'd either visualize the data of your progress or incorporating like a … broader spectrum of … moods … It kind of feels like … there are a lot of apps out there that might be doing kind of similar things that could maybe be integrated. Just like taking the best pieces of other apps and kind of like working them in would be cool. |
|  | QJ | P36: I was just gonna say … if you're trying to maybe appeal to the fun factor more, you would probably want things that felt like someone was … making real achievements, and maybe literally animation would be helpful for that. |
|  | QJ | P33: They can have a feature … to just link … like, listen to your favorite song, or you could have … certain contacts … you want to talk or call … linked in like a one press button, or … something like YouTube. |
|  | QJ | P16: So, is there anywhere in the app … where … every time you buy a pack, you can press that you bought a pack of cigarettes, just so you can see how much money you’re spending … just to give it a timeline of how much money you save that month. |

Participant ID appears before each quote for attribution.
There was 144 suggestions total, 64 for Quit Guide and 80 for Quit Journey.
QG = Quit Guide, QJ = Quit Journey.
^*^Indicates suggestion already present in app.
